# In planta interactions of a novel bacteriophage against *Pseudomonas syringae* pv. *tomato*

**DOI:** 10.1007/s00253-023-12493-5

**Published:** 2023-04-19

**Authors:** Dimitrios Skliros, Polyxeni Papazoglou, Danai Gkizi, Eleni Paraskevopoulou, Pantelis Katharios, Dimitrios E Goumas, Sotirios Tjamos, Emmanouil Flemetakis

**Affiliations:** 1 Laboratory of Molecular Biology, Department of Biotechnology, School of Applied Biology and Biotechnology, Agricultural University of Athens, 11855 Athens, Greece; 2 Department of Wine, Vine and Beverage Sciences, School of Food Sciences, University of West Attica, 12243 Athens, Greece; 3 Institute of Marine Biology, Biotechnology and Aquaculture, Hellenic Centre for Marine Research, 71500 Heraklion, Greece; 4 Laboratory of Plant Pathology-Bacteriology, Department of Agriculture, School of Agricultural Sciences, Hellenic Mediterranean University, 71004 Heraklio, Estavromenos Greece; 5 Laboratory of Plant Pathology, Department of Crop Science, School of Plant Sciences, Agricultural University of Athens, 1855 Athens, Greece

**Keywords:** Bacteriophage, Biological control, *Pseudomonas syringae* pv. *tomato*, *Solanum lycopersicum*, Phytopathogen, Siphovirus

## Abstract

**Abstract:**

The biology and biotechnology of bacteriophages have been extensively studied in recent years to explore new and environmentally friendly methods of controlling phytopathogenic bacteria. *Pseudomonas syringae* pv. *tomato* (Pst) is responsible for bacterial speck disease in tomato plants, leading to decreased yield. Disease management strategies rely on the use of copper-based pesticides. The biological control of Pst with the use of bacteriophages could be an alternative environmentally friendly approach to diminish the detrimental effects of Pst in tomato cultivations. The lytic efficacy of bacteriophages can be used in biocontrol-based disease management strategies. Here, we report the isolation and complete characterization of a bacteriophage, named Medea1, which was also tested in planta against Pst, under greenhouse conditions. The application of Medea1 as a root drenching inoculum or foliar spraying reduced 2.5- and fourfold on average, respectively, Pst symptoms in tomato plants, compared to a control group. In addition, it was observed that defense-related genes *PR1b* and *Pin2* were upregulated in the phage-treated plants. Our research explores a new genus of *Pseudomonas* phages and explores its biocontrol potential against Pst, by utilizing its lytic nature and ability to trigger the immune response of plants.

**Key points:**

*• Medea1 is a newly reported bacteriophage against Pseudomonas syringae pv. tomato having genomic similarities with the phiPSA1 bacteriophage*

*• Two application strategies were reported, one by root drenching the plants with a phage-based solution and one by foliar spraying, showing up to 60- and 6-fold reduction of Pst population and disease severity in some cases, respectively, compared to control*

*• Bacteriophage Medea1 induced the expression of the plant defense-related genes Pin2 and PR1b*

**Supplementary Information:**

The online version contains supplementary material available at 10.1007/s00253-023-12493-5.

## Introduction

Tomato (*Solanum lycopersicum*) cultivation is essential for many countries. *Pseudomonas syringae* pv. *tomato* (Pst) is a very important pathogen responsible for bacterial speck disease of tomatoes, occurring worldwide (Wilson et al. 2002). Characteristic symptoms caused by Pst are leaf lesions, defoliation, and fruit lesions via natural openings (stomata or unintentional wounds) that spread in the apoplastic space with a minimal systemic distribution (Gullino et al. 2009; Xin and He 2013), resulting in a significant yield loss. In nature, *P. syringae* can survive on the surface of leaves as well as in soil, and it can spread from plant to plant in environments that are favorable for it (high humidity, rain, and a comfortable temperature). The protection of crops against pathogens at all stages of plant development is a crucial component of the agricultural industry. The control of this disease depends mainly on the use of copper-based agrochemical products (Wilson et al. 2002). However, this control strategy has as a major drawback the rapid development of plasmid-mediated copper tolerance (Bender and Cooksey 1986; Cuppels and Elmhirst 1999; Griffin et al. 2019). Streptomycin has also been used to control the disease (Conlin and McCarter 1983) with antimicrobial resistance occurring in a short time frame (Scheck et al. 1996). Nowadays, the use of antibiotics for the control of bacterial pathogens in plants is not encouraged in most countries as the development and dissemination of antibiotic resistance could be a threat to human health (Sundin and Wang 2018). The lack of efficacious pesticides against Pst or host resistance drives agriculture towards the implementation of alternative disease management strategies.

Since the last decade, scientists are utilizing biocontrol agents (BCAs) to prevent bacterial speck disease in tomatoes with most of the BCAs so far being bacteria (Wilson et al. 2002; Ji et al. 2006; Durairaj et al. 2018). Another promising strategy that supplements the use of bacteria could be the utilization of bacteriophages. Bacteriophages are viruses that can infect exclusively bacteria, including phytopathogenic bacteria (Żaczek et al. 2015). They can show high host specificity and potentially high lytic activity against their main hosts (de Jonge et al. 2019). Bacteriophages considerably minimize their impact on the environment and non-target microorganisms. Thus, phages are considered to be more sustainable and safer than antibiotics usage (Batinovic et al. 2019).

*Pseudomonas* species have been at the center of attention in phage research. Several bacteriophages, members of different families, and genera from the *Caudoviricetes* class have been isolated in the past and have been used to elucidate several aspects of molecular interaction mechanisms (De Smet et al. 2017). Phage-based solutions and strategies for the control of phytopathogenic bacteria have also been designed and explored in the past (Balogh et al. 2010; Jones et al. 2012; Żaczek et al. 2015; Stefani et al. 2021). Nevertheless, a preventive strategy against Pst in planta has not yet been fully explored under greenhouse conditions.

Previously, bacteriophages have shown strong lytic efficacy in vitro and ex vivo against *P. syringae* phytopathogenic pathovars, suggesting that lytic bacteriophages could own a novel role against bacterial specks (Pinheiro et al. 2019). Nevertheless, the preventive utilization of bacteriophages to delay Pst growth and prime plants against Pst is yet to be explored. This study presents a newly reported bacteriophage against Pst and explores its lytic efficacy in vitro and its plant protective activity against Pst in tomato plants under greenhouse conditions. Our work could frame a modern, eco-friendly solution against Pst.

## Materials and methods

### Bacteria and bacteriophage isolation and biological characterization

The Pst strain (Pst 235) used in this study was isolated from tomato seedlings in a commercial plant nursery of Crete Island, Greece. The Pst 235 strain was deposited in Belgian Co-ordinated Collections of Micro-organisms under accession number LGM 32,894. Its genome has been sequenced and deposited in NCBI GenBank under accession number NZ_JADZGO000000000. For bacteriophage host range analysis, three more Pst strains were examined, two of them (Pst 237 and Pst 122, deposited in the local bacteria bank of the Hellenic Mediterranean University under accession numbers Pst 237 and Pst 122 respectively) isolated from commercial plant nurseries, as well as reference Pst strain *P. syringae* pv. *tomato* DC3000 (deposited in the Global Bioresource Center under accession number ATCC BAA-871).

The bacteriophage used in this study was isolated from soil samples collected from tomato seedlings under greenhouse conditions (Akbaba and Ozaktan 2021). Pst 235 was used as the main host and target bacterium. Phage isolation took place after enriching soil samples with King’s B medium liquid broth (2% peptone, 0.15% K_2_HPO_4_, 0.15% MgSO_4_•7H_2_O, 0.1% glycerol), enriched with 1 mL/L MgSO_4_ 1 M and 1 mL/L CaCl_2_ 1 M and Pst 235 host as described in Di Lallo et al. (2014) and incubation at 150 rpm and 30 °C overnight. After incubation, 1.5 mL of each sample was centrifuged for 5 min at 17,000 g and the supernatant was filtered (0.22 μm). The spot assay technique was used to monitor the presence of bacteriophages. Petri dishes were then placed upside down in the incubation at 25 °C. Phage plaques were picked and purified three times to ensure clonal phage stocks. The filtered phage stock was stored at 4 °C for further experimentation. The morphology of the virion was observed by transmission electron microscopy (TEM). An aliquot of the phage suspension with a titer of ~ 10^9^ plaque forming units (pfu) mL^−1^ was negatively stained with 2% w/v uranyl acetate for 2 min. Medea1 bacteriophage was observed using a JEOL (Peabody, MA, USA) TEM operated at 60 kV. The size of the virion was measured by using ImageJ software (Schneider et al. 2012). For the host range analysis, the double-layer agar technique was used, with a high titer phage stock lysate (10^9^ pfu/mL). Serial diluted large droplets of 30 μL were used in the top agar in triplicates. Evaluation of phage lysis of the bacteriophage was compared with the results taken from the main host Pst strain (Pst 235). The lytic activity of Medea1 was classified into two categories: strong activity (clear lysis with same number of phage plaques and not different plaque morphology from the main host, +  + +) and medium activity (clear plaque with faintly hazy background, + +).

### Bacteriophage growth kinetics

For monitoring bacteriophage growth, a Pst 235 liquid culture was infected with a bacteriophage aliquot at its exponential growth phase (OD_600_ = 0.2; 2 × 10^8^ colony forming units (cfu)/mL), with sampling times 5, 10, 15, 20, 25, 30, 40, 60, 70, 80, 90, 100, 120, 150, 180, 220, 240, 300, 330, 400, 430, 500, 530, 600, 630, 700, 730, and 800 min as previously mentioned for other Pst bacteriophages (Di Lallo et al. 2013) and multiplication of infection (MOI) factor being 1, meaning that the number of bacteria and free bacteriophages is 1. For monitoring phage adsorption, we generated a second bacteriophage growth curve with MOI: 0.01 and sampling times 5, 10, 15, 20, 25, 30 40, 60, 70, 80, 90, 100, and 120 min. The moment of phage addition was considered as the time *t* = 0. From the Pst/phage co-culture, a 200 μL aliquot was sampled and added in 100 μL of chloroform and centrifuged at 20,000 g for 60 s. Using the double-layer agar technique, 100 μL of each sample was plated in King’s (1.5% (w/v) agar) along with 100 μL of Pst 235 liquid culture and 3 mL King’s (0.5% (w/v) agar). The plates were incubated at 25 °C. After 1 day, the lytic plates were counted and the phage titer was calculated for each time point. Three independent assays were performed. The results were subsequently plotted to determine the phage eclipse, latent, adsorption period, and burst size.

### Biofilm disruption assay

The microtitre plate biofilm formation assay was performed according to the method of O’Toole and Kolter (1998) with modifications. Pst 235 biofilm was formed by adding 950 μL of Pst235 (OD_600_ = 0.8) and 50 μL of King’s B medium in tissue culture polystyrene 24-well microtitre plates (Costar 3524, Corning, NY, USA) at 25 °C for 7 days. After biofilm formation, 50 μL of the phage Medea1 at MOI 0, 10, 100, and 500 was added to the wells in triplicates. The plates were incubated for 3 days at 25 °C. Biofilm formation was monitored in terms of OD 540 nm, after staining with Crystal Violet solution (O’Toole and Kolter 1998), using a microplate reader (TECAN Infinity series 200 PRO, Männedorf, Switzerland) at 1 and 3 days post-bacteriophage inoculation (dpi).

### Bacteriophage and bacteria DNA extraction for next-generation sequencing

DNA extraction was performed after a PEG-8 k virion precipitation as described in Skliros et al. (2016). Briefly, viral capsids were lysed with proteinase K after treating the samples with DNase and RNase for removing the host’s DNA and RNA. DNA was extracted by using a DNA extraction kit (QIAGEN, Hilden, Germany). Illumina Ηiseq 2000 (Illumina, San Diego, CA, USA) sequencing took place according to the manufacturer’s protocols (Lifesequencing, Valencia, Spain). A genomic library was generated with an optimized size selection using magnetic bead purifications based on the standard Illumina protocol and by using a Nextera XT Library Construction Kit (Illumina, San Diego, CA, USA). Five micrograms of the extracted DNA was used for quality control with a Bioanalyzer (Agilent, CA, USA) and for the construction of a paired-end library with an insert size of 500 bp.

### Bioinformatics analysis

After the phage DNA sequencing, possible contaminated reads, primers, adapters, and 3′-, 5′- low-quality reads were trimmed off with an error rate threshold of 0.05 from the raw data. De novo assembly was conducted using the Geneious platform (www.geneious.com/ R10 version; Biomatters Ltd., Auckland, New Zealand; Kearse et al. 2012). Assembly resulted in a single contig which was considered the genome of *Pseudomonas* phage Medea1. In silico prediction of open reading frames (ORFs) was performed with the gene caller software Glimmer (http://ccb.jhu.edu/software/glimmer/index.shtml version 3.0 (Delcher et al. 1999)) using the bacterial, archaeal, and plant plastid code (translation table 11). The Rapid Annotation Subsystem Technology (R.A.S.T.; Aziz et al. 2008; Overbeek et al. 2013) and the B2Go (www.biobam.com/download-blast2go/ BioBam, Valencia, Spain) platform were used for annotation, against the non-reductant protein database and UniProt database with an E-value threshold of ≤ 10^−6^. The results from all annotators were filtered, evaluated, and combined. The presence of analogous or homologous genes and open reading frames (ORFs) among the genomes of bacteriophages Medea1, PSA1, and Lambda phage (Supplemental Table S1) was identified through the online platform CoreGenes3.5 (http://binf.gmu.edu:8080/CoreGenes3.5/) (Zafar et al. 2002) with a BLASTp threshold score of 45. Phylogenetic trees were generated using Geneious with certain parameters as previously demonstrated for dsDNA tailed phage genes (Low et al. 2019; Jones–Taylor–Thornton substitution model, nearest-neighbor-interchange model of tree interference and 100 bootstraps). A bacteriophage proteomic tree was generated with ViPtree (https://www.genome.jp/viptree/ version 1.9; Nishimura et al. 2017). The genome of bacteriophage Medea1 has been deposited in NCBI GenBank under accession number MW862109.

### Pathogen preparation

A rifampicin-resistant mutant of the Pst 235 strain was used throughout the in vivo experiments for screening solely this specific Pst strain at the microbiological assay during the in vivo study as described previously (Fousia et al. 2015). The colonies of the rifampicin-resistant mutant strain were selected in Petri dishes containing 100 μg/mL rifampicin after overnight incubation at 28 °C for 24 h. The selected colonies were cultured in liquid King’s B medium containing 50 μg/mL rifampicin and placed in an orbital incubator at 180 rpm at 28 °C for 24 h. The bacterial suspension was centrifuged at 5000 g for 10 min and resuspended in 50 mM phosphate buffer, pH 7, providing a cell concentration of 5 × 10^6^ cfu/mL in a total volume of 150 mL.

### In planta pathogenicity experiments

The in planta activity of Medea1 against Pst 235 was examined in tomato plants (cultivar monfavet) under a challenge-based experimental setup. For examining different phage application methodologies, we utilized a root drenching (RD) application method of the phage and a foliar spraying (FS) application method of the phage. A positive control of only Pst bacterium was also used. Additionally, for phage application, three control sets of treatments were prepared without Pst challenge: mock plants, a phage root drenching control (RD), and a phage foliar spraying control (FS) (Supplemental Fig. S1). Positive control plants were sprayed to run off with the Pst 235 solution but not with the phage solution. Mock plants (control plants) were watered with distilled water during the experiment. All plants were incubated under the same greenhouse conditions. A filtered (with a 0.22-μm pore filter) Medea1 phage stock aliquot (10^8^ pfu/mL) was prepared, which was diluted in a solution of ddH_2_O, CaCl_2_ 1 M, and MgSO_4_ 1 M (total volume of 4 L) with a final titer of 2.5 × 10^5^ pfu/mL. In this way, lysate containing bacterial debris and proteins were also highly diluted. The Medea1 phage solution was applied in tomato plants (6 leave stage) grown in pots containing sterilized soil (Potground, Klasmann-Deilmann GmbH, Geeste, Germany), by root drenching (PstRD) or foliar spraying (PstFS) (Supplemental Fig. S1). In each pot, four plants were grown and were root drenched with a total of 50 mL (per four plants) from the phage dilution, meaning that a total of 1.25 × 10^7^ Medea1 virions were used. Regarding foliar spraying, a total of 400 mL of the same phage aliquot solution was used, meaning that each pot with four plants was foliar sprayed approximately with 50 mL (per four plants) of 2.5 × 10^5^ pfu/mL or total 1.25 × 10^7^ Medea1 virions. After 1 day, the tomato plants assigned for the challenge experiment were sprayed to run off with Pst 235. The plants were enclosed in transparent plastic bags with 100% humidity. Plants were kept under greenhouse conditions at 20–30 °C with a 12 h photoperiod. The experiment was repeated three times. Bacterial speck symptoms were recorded after 14 days post-inoculation (dpi) with Pst. Disease severity was calculated by the percentage of leaves that showed bacterial specks to the total number of leaves per plant.

### Evaluation of the Pst 235 titer in plant leaflets

Leaflets from the top, middle, and bottom parts of four individual plants per treatment were collected at 1 and 3 dpi. Leaf discs of 1 cm diameter were cut off and a total of 8 cm^2^ of leaflets were used (8 leaflets from 4 plants, 2 leaflets/plant, 1 cm^2^/leaf). The leaf discs were sterilized in 70% ethanol for 1 min and subsequently rinsed with ddH_2_O for 30 s. Appropriate tenfold dilutions from the homogenate were plated on King’s B medium (1.5% (w/v) agar, 50 μg/mL rifampicin) for selecting the rifampicin-resistant mutant. The plates were incubated at 28 °C for 48 h whereas the colony-forming units were counted.

### RNA isolation and quantitative PCR for gene expression

Leaflets from the top, middle, and bottom parts of three plants from the virus treatments (sprayed and drenched) and control plants (pathogen and mock-inoculated plants) were sampled at 1 and 3 dpi. The leaflets were ground to a fine powder using an autoclaved mortar and pestle, in the presence of liquid nitrogen and stored at − 80 °C. Total RNA from each sample was extracted using Nucleozol™ Reagent (Macherey–Nagel, Düren, Germany), according to the manufacturer’s instructions. The RNA samples were treated with TURBO™ DNAse (Thermo Fisher, Waltham, MA, USA), and their concentration was measured on a Nanodrop ND-1000 spectrophotometer (Saveen Werner, Malmö, Sweden). First-strand cDNA was synthesized using SuperScript II (Invitrogen, Waltham, MA, USA), following the manufacturer’s instructions. The expression levels of the *Pin2* gene, which expresses a component of the auxin efflux carrier (Herman et al. 2008) and the *PR1b* gene, which expresses a pathogenesis-related protein involved with jasmonic acid metabolism (Block et al. 2005) were studied. Both genes are involved in a wide range of abiotic and biotic plant defense systems in plants. For estimating their relative expression, we used the following primer sequences: for *PR1b* (M69248), forward 5′-GGTCGGGCACGTTGCA-3′ and reverse 5′-GATCCAGTTGCCTACAGGACATA-3′ (Block et al. 2005); and for *Pin2* (JN091682), forward 5′-TGATGCCAAGGCTTGTACTAGAGA-3′ and reverse 5′-AGCGGACTTCCTTCTGAACGT-3′ (Herman et al. 2008). The actin gene and the ubiquitin gene were used as housekeeping genes. The actin gene expression levels were detected using the primer pair: forward 5′-TTGCCGCATGCCATTCT-3′ and reverse 5′-TCGGTGAGGATATTCATCAGGTT-3′ (Herman et al. 2008). The ubiquitin gene expression levels were detected using the primer pair: forward 5′-GTGTGGGCTCACCTACG-3′ and reverse 5′-ACAATCCCAAGGGTTGT-3′. The expression levels of the phage tail virus gene and endolysin gene were detected after designing in silico primers using Geneious platform (R10 version; Biomatters Ltd., Auckland, New Zealand; Kearse et al. 2012). The primer sequences were the following: forward 5′-CGCAATCATCCTCGACAACG-3′ and reverse 5′-ACCTGCCAGCTTGTAGAAGG-3′, and for monitoring the expression of phage endolysin gene, the primers pair sequences for forward 5′-CAGCAGTTGGGATACGAGGG-3′ and for reverse 5′-CACGGTATTTGGAGCCATCG-3′. qPCR was performed on a StepOnePlus™ Real-Time PCR System (Applied Biosystems, Foster City, CA, USA) using SYBR Select Master Mix (Applied Biosystems, Austin, TX, USA), gene-specific primers at a final concentration of 0.2 μM each, and 1 μL of the cDNA as template. Primer specificity and formation of primer dimers were monitored by dissociation curve analysis. PCR cycling started at 95 °C for 10 min, followed by 40 cycles of 95 °C for 15 s and 60 °C for 1 min. Housekeeping genes were used to normalize cDNA templates and compute the relative transcript levels for each gene, which were calculated as (1 + E)^−ΔCt^. ΔCt was calculated as (Ct_X_–Ct_H_), whereas Ct_X_ corresponds to the Ct of the targeted gene, and Ct_H_ is the geometric mean of the two housekeeping genes’ Cts. PCR efficiency (E) for each amplicon was calculated by applying the linear regression method to the log (fluorescence) per cycle number data, using LinRegPCR software (Ramarkers et al. 2003).

### Plant’s DNA extraction and relative estimation of DNA abundance

Leaflets from three plants were collected simultaneously for gene expression analysis and estimation of DNA abundance for each replicate and were ground to a fine powder using an autoclaved mortar and pestle. A solution based on SDS (sodium dodecyl sulfate) was then added. More specifically, the pellets were resuspended in 0.2 mL of DNA extraction buffer (150 mM Tris–HCL (pH 8.0), 25 mM EDTA, 20 g SDS/L, 10 mg/mL proteinase K, RNAse 30 μg/mL, 2% β-mercaptoethanol) and incubated for 2 h at 65 °C. After heat inactivation of the proteinase K for 10 min at 95 °C, the tubes were cooled to 4 °C and centrifuged for 10 min at 17,000 g. The DNA was precipitated at 4 °C in the presence of isopropanol for 16 h. The DNA was then centrifuged at 18,000 g for 20 min and purified 2 times using a solution of chloroform and phenol (PIC, phenol:chloroform:isoamylic alcohol 25:24:1). The DNA was then precipitated using 100% ethanol and hydrated with 75% ethanol before redissolving in water. Ten nanograms of DNA per sample was used for the estimation of the DNA abundance of both bacteria and phage DNA. Primers used for estimating DNA abundance of Pst were targeting *hrpZ* gene of Pst, which encodes for a *P. syringae-*specific harpin protein (Zaccardelli et al. 2005), with primer sequences being forward F-5′-GAACGAGCTGAAGGAAGACA-3′ and reverse 5′-CAGCCTGGTTAGTCTGGTTA-3′. For estimation of bacteriophage DNA, a set of primers was designed targeting the phage tail coding DNA sequence (CDS) with forward being 5′-GTCAAGGCTCCGTGGAAGTA-3′ and reverse 5′-TCTTCTTGCCGTCTGTCTCG-3′. Relative estimation was calculated as described in RT-qPCR section by using as a housekeeping gene the plant’s 23S rRNA fragment, which was highly abundant in the isolated DNA and used for normalization with primers being forward 5′-GGGCGACTGTTTACCAAAAA-3′ and reverse 5′-TTACCCGACAAGGAATTTCG-3′, according to Tameshige et al. (2013).

### Statistical analysis

Data on Pst titer, disease severity, DNA abundance, and gene expression analysis were analyzed and visualized with Sigma Plot SigmaPlot version 14.0 (Systat Software, Inc., San Jose, CA USA, www.systatsoftware.com). All data passed normality and equal variance tests, except disease severity data. One-way ANOVA statistical analysis was used for identifying statistically significant differences. When a statistically significant difference (*p* < 0.05) was obtained for treatments, a post hoc Student’s *t*-test followed. For disease severity data, ANOVA on ranks with a post hoc Dunn’s method was used.

## Results

Isolation and characterization of *Pseudomonas* bacteriophage Medea1.

**Fig. 1:**
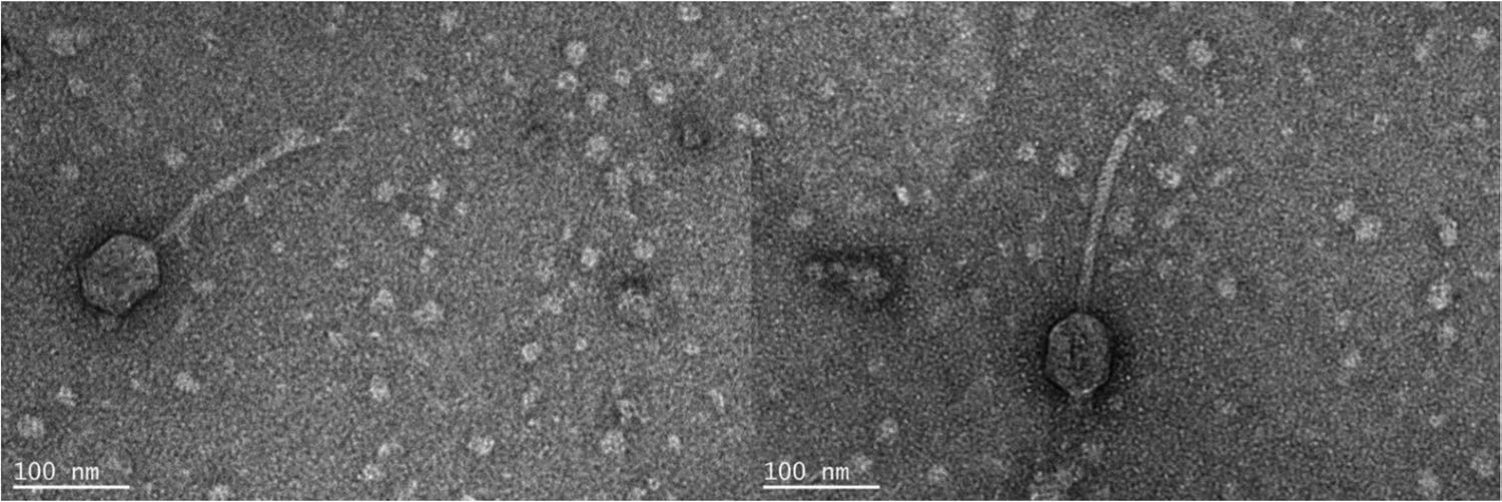
Transmission electron micrograph of bacteriophage Medea1 showing a virion with a long non-contractile tail typical of siphovirus morphotype

**Fig. 2:**
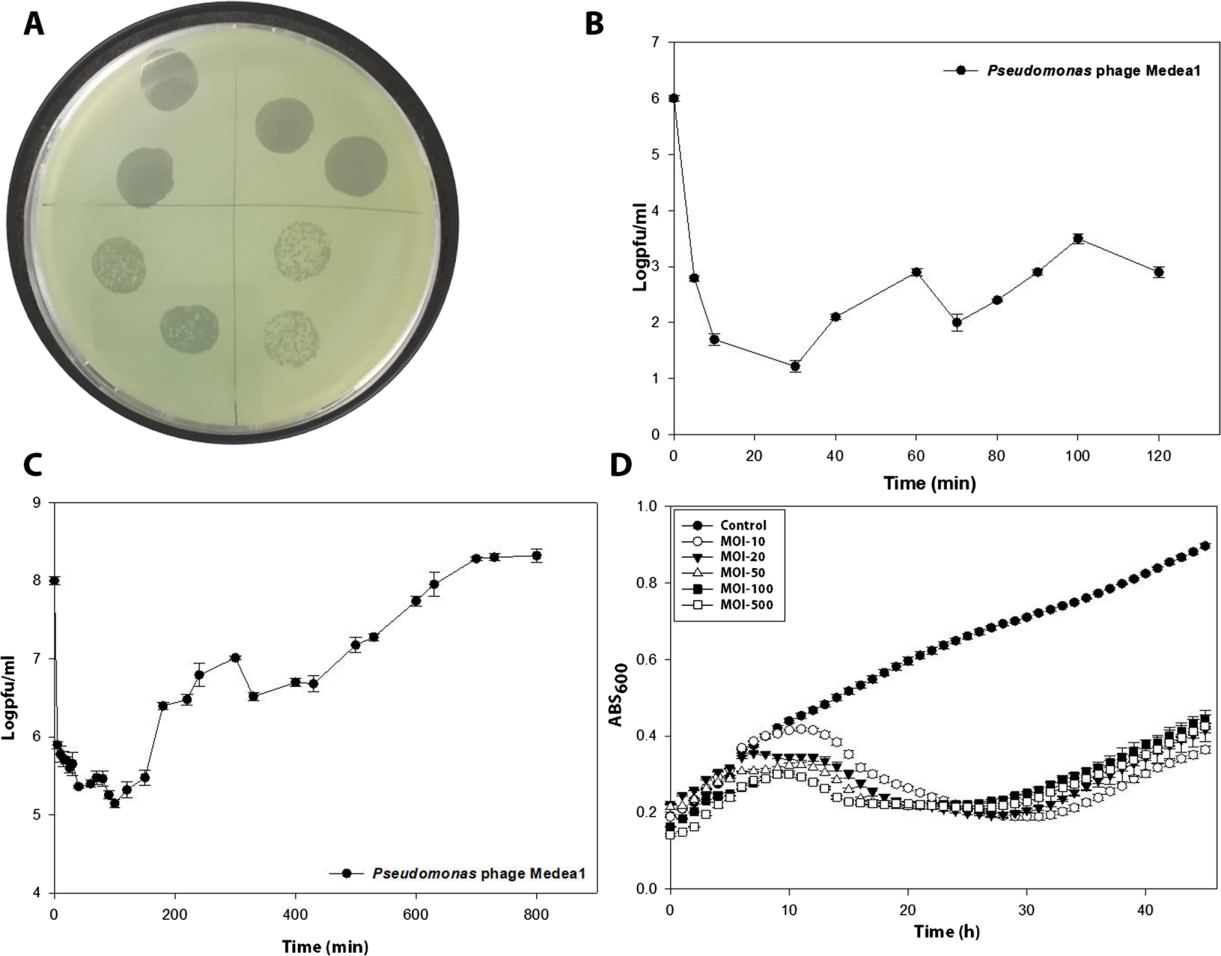
Biological characteristics of bacteriophage Medea1. **A** Spots with sequential dilutions of phage stock. **B** Phage-growth curve with MOI: 0.01 of the first 120 min to calculate 95% phage adsorption time. **C** Two-step growth curve of bacteriophage with MOI: 1. **D** Growth kinetics of the main host (Pst 235) with the addition of bacteriophage Medea1 in different MOIs (SE ±)

**Fig. 3:**
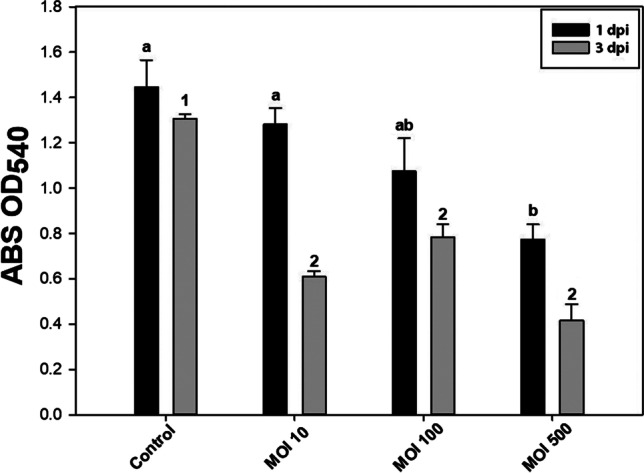
Effect of Medea1 *Pseudomonas* phage on biofilm of *Pseudomonas syringae* pv. *tomato* depending on time and MOI of application 1 day post-inoculation (1 dpi) and 3 days post-inoculation (3 dpi) with the bacteriophage. Different letters or numbers indicate statistically significant differences between treatments. Comparative statistics were conducted separately for each dpi treatment (one-way ANOVA, post hoc analysis for *p* ≤ 0.05 with Student’s *t*-test, SE ±)

The bacteriophage isolation process resulted in a bacteriophage with strong lytic activity against Pst 235. TEM revealed a bacteriophage with a siphovirus morphotype, a tail length of 156.05 nm (SE ± 4.67; *n* = 10), and a capsid length of 71.66 nm (SE ± 2.13; *n* = 10) (Fig. 1). Host range experimentation showed that bacteriophage Medea1 analysis can lyse efficiently other local clinical isolates of Pst, as well as the reference strain DC 3000, although phage plaques were faintly hazy (Table 1). Serial dilutions did not show differences in the number of phage plaques among strains. Biological characterization of the bacteriophage included a phage morphology characterization, step growth curve, in vitro lytic efficacy, and antibiofilm activity (Fig. 2, Fig. 3). Bacteriophage spot assay formed clear lysis zones and round phage plaques in double-layer culture (Fig. 2A). Ninety-five percent of the virions can adsorb to their main host within 10 min, although the total multiplication time of the bacteriophage was estimated at 200 min (Fig. 2B, Fig. 2C).

**Table 1:** Host range of bacteriophage Medea1 against different *Pseudomonas syringae* pv. *tomato* strains including reference strain Pst DC3000. Strong activity (+ + +) and medium activity (+ +) are demonstrated as described in materials and methods section

Bacterial host strain	Phage Medea1 lysis evaluation	Reference
Pst 235 (main host)	** + + + **	This work
Pst 237	** + + + **	Not available
Pst 122	** + + + **	Not available
Pst DC3000	** + + **	Buell et al. 2003

Additionally, the phage growth curve revealed a burst size of approximately ~ 30 phage particles/infected cell. In vitro host lysis experiment showed a bacteriophage that can suppress bacterial host to initial titer for more than 20 h. Thus, we proceeded with the in vitro anti-biofilm activity of the bacteriophage (Fig. 3). A twofold reduction of the Pst biofilm was observed 1 day after the addition of the phage at a MOI of 500. The same twofold disruption remained at 3 dpi for MOI 500, but also the same reduction was observed for MOI 10 and MOI 100, suggesting that time and MOI are crucial factors for biofilm disruption. Nevertheless, a plateau of reduction of 3.5-fold was observer at OD_540_ = 0.4.

These interesting results led us to continue with a genomic characterization of bacteriophage Medea1, for studying the molecular features of the bacteriophage. After bacteriophage nucleic acid isolation, at first, we were able to conclude by using DNAse and RNAse enzymes that indeed this bacteriophage harbors dsDNA. Illumina DNA sequencing followed, which resulted in 4 mil reads with an average length of 250.7 bp. The assembly process revealed a single contig of 58.919 bps and a GC content of 57.9%. In silico annotation process revealed that at least 86 CDS from which we were able to identify 29 and 57 were considered hypothetical proteins (Supplemental Fig. S2). The molecular characteristics and the proposed taxonomy of the phage are presented in Table 2**.** We did not detect any genes for t-RNAs. Nevertheless, detailed genome annotation showed the presence of an integrase protein (Supplemental Table S1).

**Table 2:** Brief characteristics of the newly reported *Pseudomonas* bacteriophage Medea1

Bacteriophage name	Accession number	Phylogeny	Main host	Sequence length	GC (%) percentage	ORFs
*Pseudomonas* phage Medea1	MW862109	*Viruses; Duplodnaviria; Heunggongvirae; Uroviricota; Caudoviricetes*	*Pseudomonas syringae* pv. *tomato* Pst235	58,919 bp	58.1%	86

### Comparative genomics of Medea1 phage

**Fig. 4:**
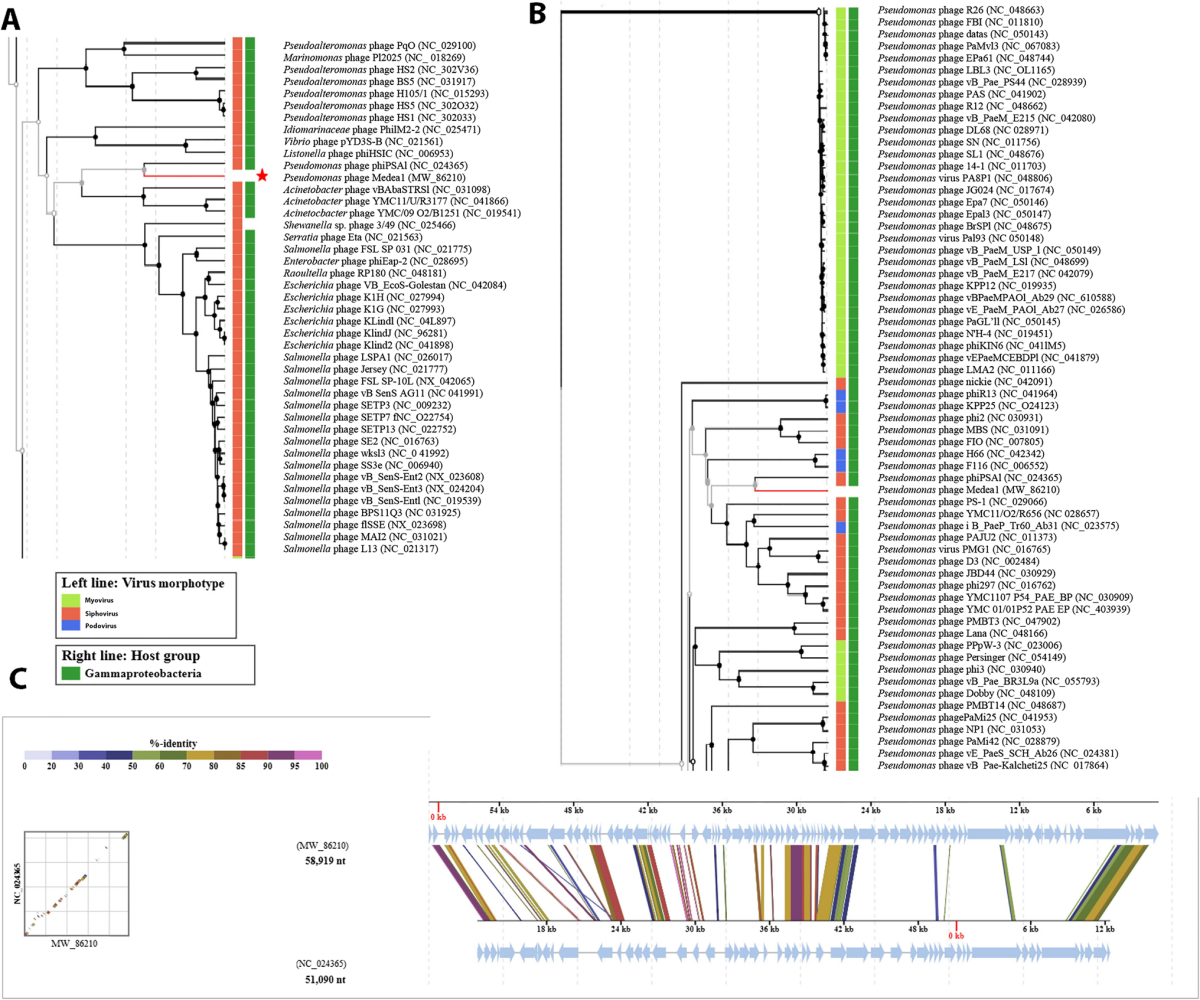
Viral proteomic tree generated with VipTree online software of *Pseudomonas* phage Medea1 and its closest relatives with (**A**) a different host genus and (**B**) the same host genus. Legend corresponds to viral morphotype. Also, (**C**) a pairwise genome sequence alignment of *Pseudomonas* phage Medea1 and *Pseudomonas* phage PSA1 is shown

Blastn algorithm did not detect any significant whole-genome similarities among *Pseudomonas* phage Medea1 and other bacteriophages. Nevertheless, VipTree revealed that the proteome of Medea1 bacteriophage has the highest similarity with the *Pseudomonas* bacteriophage PSA1, a lysogenic bacteriophage infecting *P. syringae* pv. *actinidae* (Di Lallo et al. 2013). These two phages also have proteome similarity with *Acinetobacter* phages vB_Abas_TRS1, YMC11/11R3177, and YMC/09/02/B1251 (Fig. 4A, C). When studying only *Pseudomonas* phages, a proteomic tree was generated showing similarity with *Pseudomonas* phages phi2, MD8, and F10 (Fig. 4B). Specific well-established amino acid sequences, which have been used in the past as phylogenetic markers to better understand its bacteriophage genomic origins, were also analyzed. By utilizing different protein markers, we can conclude diverse evolutionary aspects of a bacteriophage (Skliros et al. 2022).

Hence, we studied the tail tape measure protein of tailed *Pseudomonas* bacteriophages with siphovirus morphotype, responsible for the tail length and the DNA transit to the cell cytoplasm during inoculation, to construct a phylogenetic tree of the known siphovirus morphotype *Pseudomonas* phages (Supplemental Fig. S3). Results confirmed the close relationship of Medea1 with PSA1. Terminase large subunit is also a key component of the DNA packaging machine found in bacteriophages. Phylogeny study revealed the relationship of that protein with *Pseudomonas* phages MP48, JBD5, and JD024. Interestingly, the integrase amino acid sequence revealed a different story. Phage integrases are enzymes that mediate unidirectional site-specific recombination between two DNA recognition sequences, the phage attachment site, and the bacterial attachment site. Medea1’s integrase groups with bacteriophages vb_PaeS_PMG1 and YMC11/02/r656 show diverse evolutionary events, which took place to form the genome of Medea1.

### Evaluation of Medea1 plant protective activity against Pst in tomato plants

**Fig. 5:**
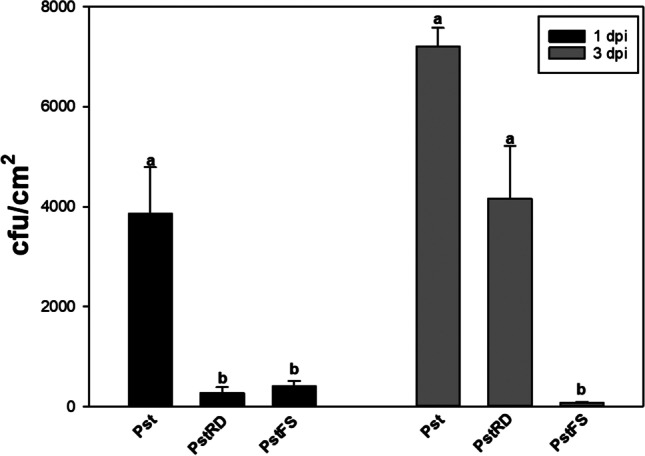
Microbiological quantification of Pst 235 bacteria 1 and 3 dpi after the initial application of Pst. Bars show bacterial titer per cm.^2^ of plant leaves. Pst inoculation without the bacteriophage, PstRD for root drenching application with the bacteriophage, PstFS for foliar spraying application with the bacteriophage. Different letters indicate statistically significant differences between treatments. (SE ± , *p* ≤ 0.05)

To test the plant protective efficacy of bacteriophage Medea1 against Pst 235 in vivo, tomato plants were treated with Medea1 24 h before Pst infection. Quantification of the *P. syringae* pv. *tomato* on the leaflets of tomato plants was done 1 and 3 dpi. The results showed that Medea1 significantly decreased the population of Pst in tomato leaflets. At 1 dpi, phage application by root drenching and foliar spraying decreased the Pst population by 20-fold, when compared to controls. At 3dpi, there was no statistically significant difference between Medea1 root drenched plants and controls. On the other hand, the Pst population was found 60-fold reduced in the Medea1 spray plants compared to the controls and the Medea1 root drenched plants (Fig. 5).

**Fig. 6:**
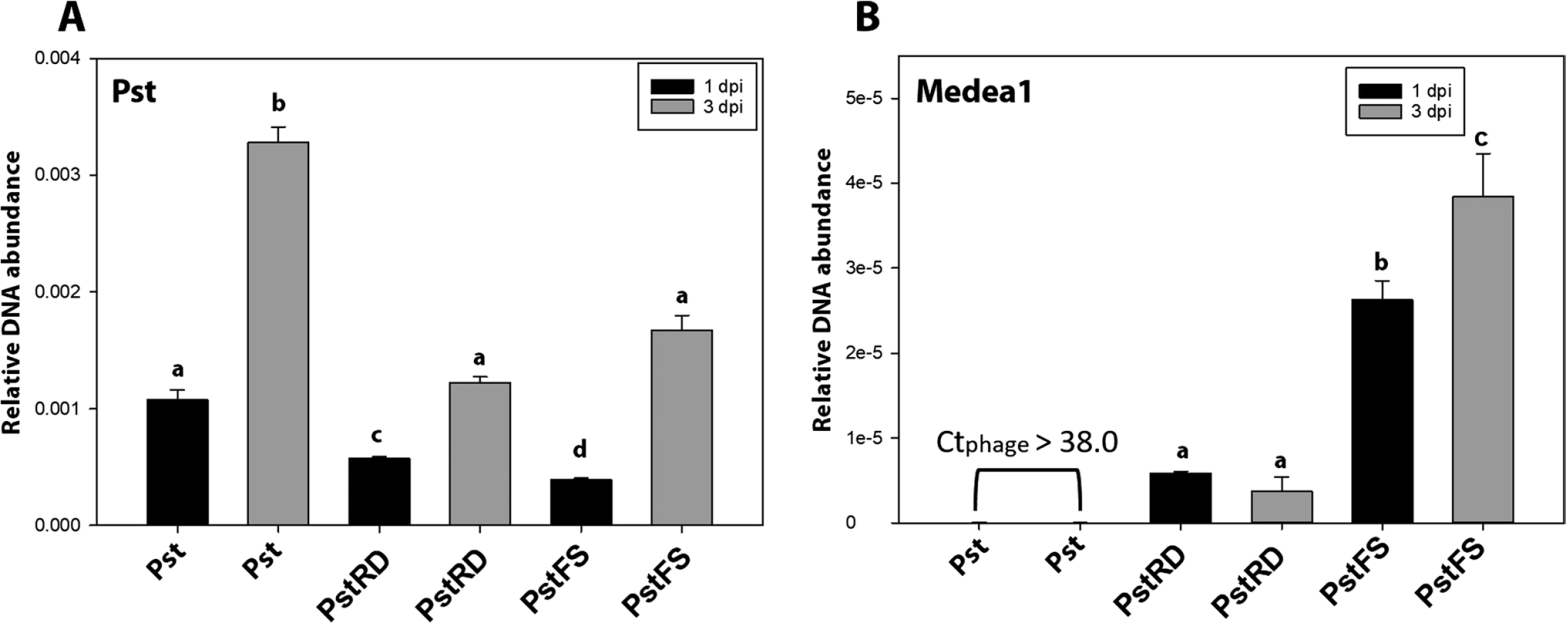
Relative DNA quantification of *Pseudomonas syringae* (**A**) and bacteriophage Medea1 (**B**) from tomato leaves 1 day post-inoculation (1 dpi) and 3 days post-inoculation (3 dpi) with Pst. Pst for inoculation without the bacteriophage, PstRD for root drenching application with the bacteriophage, PstFS for foliar spraying application with the bacteriophage. Different letters indicate statistically significant differences between treatments (SE ± , *p* ≤ 0.05)

Additionally, RT-qPCR was used for the relative quantification of Pst and Medea1 populations in tomato leaflets (Fig. 6). At 1 dpi, the Pst population in the Medea1-treated plants was twofold lower than controls (Fig. 6A). Similar results were observed at 3 dpi even if Medea1 and Pst relative DNA was increased in all treatments (Fig. 6A). Additionally, the Medea1 relative DNA content was higher in the foliar application compared to root drenching, increasing over time in the foliar sprayed plants (Fig. 6B).

**Fig. 7:**
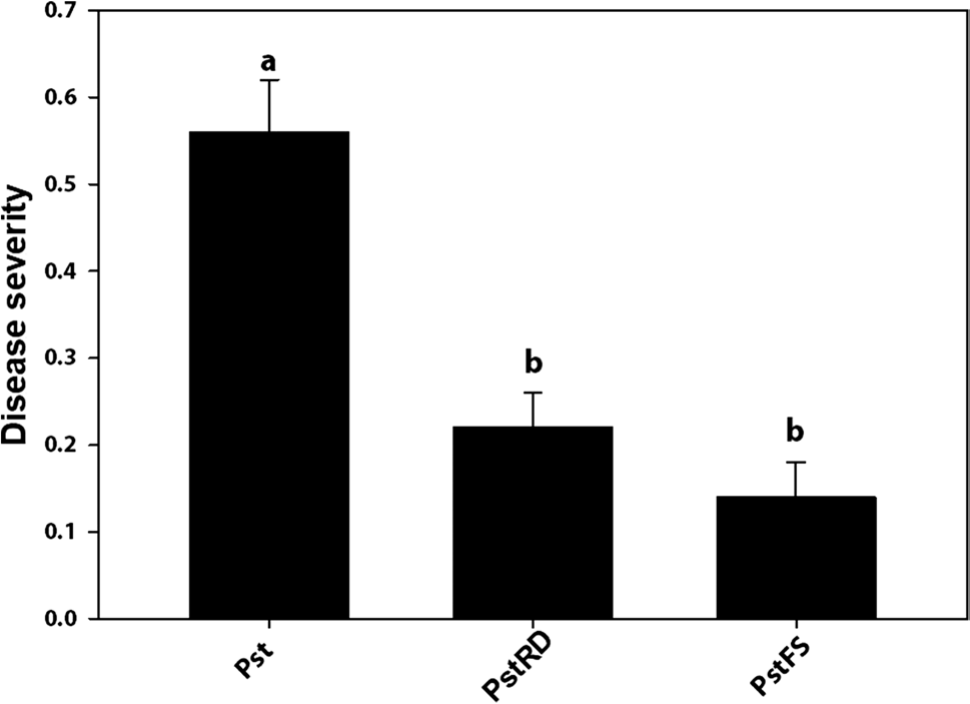
Disease severity expressed as average symptoms per plant 14 days post-inoculation with Pst. Pst for application with bacteriophages, PstRD for root drenching application with the bacteriophage, PstFS for foliar spraying application with the bacteriophage. Different letters indicate statistically significant differences between treatments (SE ± , *p* ≤ 0.05)

Plant symptoms were monitored at 14 dpi. Results showed a statistically significant difference in plant symptoms between controls and Medea1-treated plants. The disease severity was ca sixfold higher in controls compared to Medea1-treated plants. There was no difference in disease severity between the root drenching and the foliar spray application method of Medea1 (Fig. 7).

Expression analysis of *Pin2* and *PR1b* tomato genes .

**Fig. 8:**
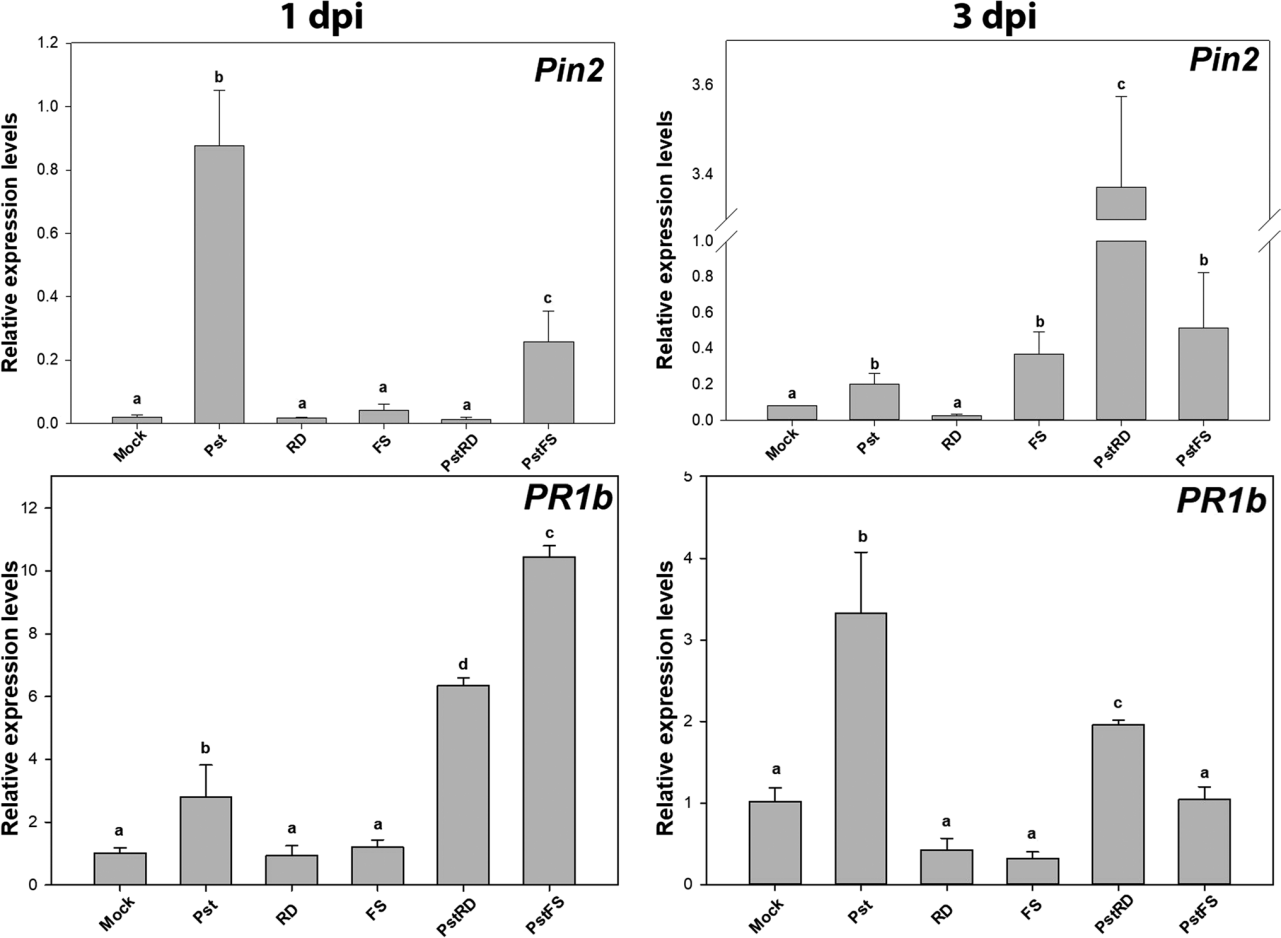
Relative expression level analysis of *Pin2* and *PR1b* genes from tomato leaves 1 day post-inoculation (1 dpi) and 3 days post-inoculation (3 dpi). Pst for inoculation without bacteriophage, RD for root drenching application with the bacteriophage without Pst, FS for foliar spraying application with the bacteriophage phage without Pst, PstRD for root drenching application with the bacteriophage, including PstFS for foliar spray application with the bacteriophage including Pst. Different letters indicate statistically significant differences between treatments (SE ± , ANOVA *p* ≤ 0.05 post hoc Student’s *t*-test)

Phytopathogenic bacteria and biocontrol agents have been shown to induce plant defense mechanisms, including the abscisic acid (ABA) metabolic pathway and the salicylic acid pathway. That is why we chose to quantify *Pin2* and *PR1b* transcripts, in two different time points, involved in different metabolic responses of defense by tomato plants (Fig. 8). Results showed a statistically significant sixfold induction of the *Pin2* gene compared to the control when only Pst was applied. Compared to control, bacteriophage applications without Pst inoculum and root drenching application with inoculum, in 1 dpi, did not exhibit statistically significant differences. In contrast, the application of the bacteriophage with the foliar spraying methodology induced *Pin2* transcript accumulation ca threefold compared to the control. These patterns were slightly different 3 days post-infection, whereas *Pin2* remained upregulated in all treatments except the root drenching application with the bacteriophage during the absence of inoculum. More specifically, the application of Pst retained the upregulation of *Pin2* with a threefold statistically significant difference compared to the control. Interestingly, the application of the bacteriophage by foliar spray without Pst inoculum also showed a threefold statistically significant upregulation of *Pin2* transcripts. A statistically significant 34-fold upregulation was also monitored during root drenching application of the bacteriophage when including the Pst inoculum.

On the other hand, the *PR1b* gene was induced in all Pst application methods compared to the control, and in both 1 and 3 dpi. More specifically at 1 dpi, Pst inoculum application showed a 3.5-fold upregulation; Pst inoculum including foliar spray bacteriophage application showed a 20-fold upregulation compared to control; root drenching application with the bacteriophage also showed a 12-fold upregulation. A slightly different pattern was observed in 3 dpi. Pst inoculum application retained a 3.5-fold upregulation of *PR1b*, while inoculum including foliar spray bacteriophage application did not show any difference from the control. Finally, inoculum and root drenching application with the bacteriophage showed a ca twofold upregulation compared to controls. The applications including only bacteriophage Medea1 did not show any difference from the controls when studying this transcript. These results show that the presence of bacteriophage could delay the expected induced expression of the *Pin2* gene, but at the same time reprogram the plant leaves to express the *PR1b* gene earlier in both application methods examined here.

Finally, to monitor bacteriophage lytic activity, we studied the relative transcript levels of phage tail protein and endolysin genes. Bacteriophage transcripts were only significantly detectable at 3 dpi when the Pst inoculum was also applied. A twofold statistically significant difference between foliar spray application and root drenching method was detected (Supplemental Fig. S4), suggesting an active role of the bacteriophage Medea1 during infection with Pst.

## Discussion

The lytic efficacy of bacteriophages against bacteria is being thoroughly studied over the last years due to their potential of reducing pathogenic bacterial populations in phytopathology (Stefani et al. 2021). Biocontrol agents, such as bacteriophages, have been widely proposed for several phytopathogenic bacterial, such as *Xyllela* sp., *Xanthomonas* sp., *Pseudomonas savastanoi*, and *P. syringae*, especially under greenhouse conditions (Tarakanov et al. 2022; Zhang et al. 2022; Holtappels et al. 2021; Clavijo-Coppens et al. 2021; Jones et al. 2007; Nordeen et al. 1983), whereas the conditions of bacteria-phage interactions can be controlled and regulated.

The frequent cases of *P. syringae* pv. *tomato*, which uses as host tomato plants (*S. lycopersicum*) being the causative agent of bacterial specks in Mediterranean countries, inspired us to study the possibility of utilizing the lytic characteristics of a newly isolated bacteriophage, to control the population of *P. syringae* under a challenged-design experiment in a typical Mediterranean greenhouse.

Medea1 bacteriophage represents a new genus within the *Caudoviricetes* class with a siphovirus morphotype.

The isolation and in vitro detailed characterization of the newly reported bacteriophage, namely Medea1, revealed a siphovirus morphology bacteriophage with a long replication circle and a growth suppression of the host in vitro for over 10 h. Host range analysis showed that Medea1 could lyse at least four different *Pseudomonas* strains, three of them isolated from commercial tomato nurseries. Future host range experiments with a larger collection of Pst will showcase the host spectrum of the virus. A broad host spectrum of *P. syringae* bacteriophages has been demonstrated in the past against pv. *actinidae* (Psa) (Flores et al. 2020). Our results showed also a strong antibiofilm activity of the bacteriophage, especially 3 days post-inoculation in an already formed *Pseudomonas* sp. biofilm. The Pst biofilm is important for bacterial plant pathogenicity (Ghods et al. 2015). Ni et al. (2020) demonstrated a similar successful destruction of a *P. syringae* pv. *actinidae* biofilm by using a phage cocktail. Genome sequencing and the in silico prediction of ORFs revealed a 58,919 bp genome. Whole proteome and phylogenetic analysis showed Medea1’s similarity to PSA1 and its lambdoid nature, but also the diverse genomic inheritance from different evolution niches, a mosaic phenomenon frequently stumbled upon many *Pseudomonas* phages (Kwan et al. 2006). The complete characterization of *Pseudomonas* phage Medea1 underlined its diverse genomic characteristics and its interesting genomic features, accompanied with strong lytic characteristics. The low nucleotide similarity with other *Pseudomonas* phages could direct the creation of a new bacteriophage genus, which will include bacteriophage Medea1 according to ICTV taxonomy guidelines (Krupovic et al. 2016).

### Phytopathogenic Pst can be efficiently controlled by Medea1 under greenhouse conditions

The in vitro lytic activity against Pst under both planktonic and biofilm metabolic states of the cell inspired to design an in planta challenge-based experiment, in which two different methodologies of phage pre-application were tested. Generally, the phage-based products need to be tested under different application methodologies; thus, the study of varied strategies is needed, especially under control conditions, such as greenhouses (Stefani et al. 2021). In our case, Medea1 genomic analysis revealed a CDS (QVW29150), which in silico annotation tagged it as a putative integrase protein. The application of *Pseudomonas* phages with putative temperate characteristics to control bacterial species in mammalian and non-mammalian models has been proposed in the past (Chung et al. 2012) with positive outcomes. However, due to their potential ability to integrate their genome in hosts, applications of temperate bacteriophages should be dealt with skepticism. In our work, we have used a putative temperate phage to prevent bacterial infection in plants in a controlled and contained environment. The main purpose is that by doing so, we could investigate the molecular mechanisms that are involved in phage-host interactions in an in vivo system. Further experiments utilizing potential temperate bacteriophages, like Medea1, as models for exploring their potential of integrating their genome into their host’s chromosome followed by detailed phenotypic characterization of the lysogenized bacteria, will significantly enhance our understanding of their apparent dual nature, and safely evaluate their potential as biological control agents especially nowadays where the ability of engineering phages using gene editing tools like CRISPR/Cas13 is feasible (Adler et al. 2022). This work could set the stage for comparative in planta works among lytic and lysogenic bacteriophages against Pst. In other plant species, such as *Brassica*, the inclusion of a smart-designed phage cocktail in the irrigation system of the plants against *Xanthomonas campestris* pv. *campestris* showed highly reduced bacterial symptoms in plants, especially when phage titer exceeded 10^9^ pfu/mL (Holtappels, et al. 2022). Plant nutrient uptake via the xylem could in fact be a viable option for distributing anti-bacterial agents, such as bacteriophages in plant organs. In our case, we phage-treated the plants 1 day prior to inoculation with Pst with two different strategies: (i) by root drenching the plants with a phage-based solution and (ii) by foliar spraying the grown plants. Both strategies resulted in decreased bacterial load compared to control conditions, even 3 days post-infection with the host. Corresponding results were obtained from the RT-QPCR methodology where we quantified the phage host’s DNA. However, phage DNA showed a relative increase over time. Plant symptoms were decreased significantly 14 days post-inoculation with Pst, indicating the efficacy of Medea1 as a prophylactic treatment. Genes encoding for phage tail protein and endolysin protein are known to be transcribed during the latest stages of bacteriophage infection (Ni et al. 2021; Luke et al. 2002). By monitoring the relative expression of these genes of Medea1 bacteriophage on plant leaves, we concluded that the lytic activity of the bacteriophage had a positive increase related to time, especially in foliar spray application method which phage demonstrated a stronger lytic activity. On the leaf surfaces, Pst is usually located in the grooves of the veins and at the base of trichomes. Within a few millimeters of the initial infection sites, Pst infection is frequently contained and does not spread to other areas of the plant (Preston 2000). Pst strains often follow two lifestyles that are related to a successful disease outcome. At first, on a healthy plant’s surface, bacteria go through an initial epiphytic phase, and when they enter the plant through natural openings or unintentional wounds, they go through an endophytic phase in the apoplastic space (Xin and He 2013). The recognition of specific translocated proteins in a resistant plant cell causes the hypersensitive reaction, a rapid defense reaction of the plant that is characterized by programmed cell death (Boureau et al. 2020). Taking into consideration the infection physiology of Pst, the decreased populations monitored in our study in leaves of both root drenching treatment and foliar spray application possibly work against both epiphytic and endophytic lifestyles. Additionally, our results regarding the disease severity of Pst are in accordance with similar in planta studies, demonstrating the lytic efficacy of diverse bacteriophages under controlled conditions (Pinheiro et al. 2019; Holtappels et al. 2022). The use of a well-studied phage cocktail with strong lytic characteristics, instead of a mono-phage application, could potentially reduce Pst populations even more and also avoid or delay potential phage resistance (Holtappels, et al. 2022).

### The in planta Medea1- Pst interaction can positively reprogram the plant defense mechanisms

It is a well-established fact that bacteriophage proteins, such as major capsid protein or tail tape measure protein, are proteins derived from bacteria (Krupovic and Koonin 2017). Thus, plants could be able to recognize these proteins and induce genes involved in defense mechanisms (Bol, 2008; Weber and Bujarski 2015). The plant hormones abscisic acid (ABA) and jasmonic acid (JA) play a predominant role in the conversion of environmental signals into changes in plant gene expression and are regulated by *Pin2* gene expression upon a biotic stress, such as a phytopathogenic bacteria (Herman et al. 2008). The accumulation of ABA and JA has been described for several plant species, including potato, tomato, and tobacco (Sanchez-Serrano et al. 1991; Peña-Cortés and Willmitzer 1995). Upregulation of the *Pin2* gene is a result of a pathogen recognition and preparation of hormone regulation mainly reflected from ABA accumulation, which is considered a frontline defense at the early stages of infection by the mediation of stomatal closure against invaders or induction of callose deposition (Gupta and Roy 2018). In our case, Pst treatments and phage foliar spray treatments showed an immediate transcript accumulation of the *Pin2* gene, even 1 day post-infection, while on 3 days post-infection also the phage root drenching methodology showed gene induction. It is well known that plant biocontrol agents, such as fungi and bacteria, can trigger plant defense against their invasions (Niu et al. 2012; Gkizi et al. 2016; Fatouros et al. 2017). Thus, it is natural to hypothesize that bacterial-related proteins, such as phage proteins, could have a similar effect on plant recognition and subsequently plant metabolic reprogramming. Interestingly, bacteriophage usage by foliar spray without Pst inoculum induced *Pin2* gene 3 dpi showing that plants could recognize phage proteins as “intruders” potentially activating its ABA-induced defense system. The presence of bacterial lipopolysaccharides (LPS) in the plant’s microenvironment could also protect against phytopathogenic bacteria with no growth negative effects (Newman et al. 2022). Phage lysates of Gram-negative bacteria carry LPS from their dead hosts (Szermer-Olearnik and Boratyński 2015), although in our case the phage stock solution was highly diluted prior to application. It is difficult to establish which lysate components triggered the upregulation of *Pin2* during phage foliar spray application.

Additionally, pathogenesis-related (PR) proteins are key elements of biotic defense mechanisms and are activated in response to pathogen invasion as well (Safaie-Farahaniand and Taghavi 2017). They are regulated by *Pr* genes allosterically which results in the production of several proteins, peptides, or compounds which are toxic to pathogens or prevent pathogen infections (Wang et al. 2019). PR-1 proteins are considered mainly secreted and accumulated in the extracellular/apoplastic space facilitated by means of their N-terminal secretion peptide (Aydın Akbudak et al. 2020). The pathogenesis-related protein 1 (PR-1) gene family plays important roles in plant metabolism in response to biotic and abiotic stresses. *PR1* gene expression is induced in response to a variety of pathogens and is a useful molecular marker for the salicylic acid defense pathway and the systemic acquired resistance response (Herman et al. 2008). Expression of this gene is salicylic-acid responsive and acts secondary, after the involvement of ABA in pre- and post-invasive immune responses. Our results showed that phage-treated plants induced the *PR1b* gene earlier (1dpi) and in higher levels relatively, than the positive control treatment with Pst. That was not the case 3 days post-inoculation with Pst, whereas positive control treatments with Pst surpassed the relative transcript levels, meaning that these plants recognized the Pst invasion belatedly, subsequently having a later relative response of the salicylic acid pathway. Being able to induce *Pr1* genes early could prove pivotal for the plant’s survivability.

The isolation and detailed characterizations of bacteriophages in vitro can reveal interesting and important attributes, which could prompt us to resourcefully design novel antibacterial strategies. Here, we analyze a newly reported *Pseudomonas* bacteriophage with strong antibiofilm activity, which could be assigned to a new genus. Utilizing prophylactic its lytic nature under greenhouse conditions shows that not only is able to reduce the bacterial load in planta, but also enhances the plant’s defense responses by inducing earlier and much stronger the salicylic acid defense pathway, a phenomenon which possibly resulted in significantly reduced bacterial specks. Additionally, we are undergoing a complex set of experiments for studying the efficacy of potential phage genome integration and the detailed phenotypic characterization of lysogenic bacteria to explore the safety of Medea1 and Medea1-like phages. The precautionary irrigating or spraying strategy with phage-based products in tomato plants could complement a holistic antibacterial strategy in modern greenhouses. Future works including the continuous application of phage-based products could show more promising results.

## Supplementary Information

Below is the link to the electronic supplementary material.

Supplementary file1 (PDF 1123 KB)

## Data Availability

All data presented in this work are available upon request. All bacterial and bacteriophage samples are also available upon request. The Pst 235 strain is deposited in Belgian Co-ordinated Collections of Micro-organisms under accession number LGM 32894. Pst 237 and Pst 122 are deposited in the local bacteria bank of the Hellenic Mediterranean University under accession numbers Pst 237 and Pst 122 respectively. Reference Pst DC 3000 is deposited in the Global Bioresource Center under accession number ATCC BAA-871. The Pst strain (Pst 235) used in this study has been sequenced and deposited in NCBI GenBank under accession number NZ_JADZGO000000000. The *Pseudomonas* phage Medea1 has also been sequenced and deposited in NCBI GenBank under accession number MW86210.

## References

[CR1] Akbaba M, Ozaktan H (2021). Evaluation of bacteriophages in the biocontrol of Pseudomonas syringae pv syringae isolated from cankers on sweet cherry (Prunus avium L) in Turkey. Egypt J Biol Pest Control.

[CR2] Akbudak MA, Yildiz S, Filiz E (2020). Pathogenesis related protein-1 (PR-1) genes in tomato (Solanum lycopersicum L): bioinformatics analyses and expression profiles in response to drought stress. Genomics.

[CR3] Aziz RK, Bartels D, Best AA, DeJongh M, Disz T, Edwards RA, Formsma K, Gerdes S, Glass EM, Kubal M, Meyer F, Olsen GJ, Olson R, Osterman AL, Overbeek RA, McNeil LK, Paarmann D, Paczian T, Parrello B, Pusch GD (2008). The RAST Server: rapid annotations using subsystems technology. BMC Genom.

[CR4] Balogh B, Jones J, Iriarte F, Momol M (2010). Phage therapy for plant disease control. Curr Pharm Biotechnol.

[CR5] Batinovic S, Wassef F, Knowler SA, Rice DT, Stanton CR, Rose J, Tucci J, Nittami T, Vinh A, DrummondCG,  GRS (2019). Bacteriophages in natural and artificial environments. Pathogens.

[CR6] Bender CL, Cooksey DA (1986). Indigenous plasmids in Pseudomonas syringae pv tomato: conjugative transfer and role in copper resistance. J Bacteriol.

[CR7] Boureau T, Routtu J, Roine E, Taira S, Romantschuk M (2002). Localization of hrpA-induced Pseudomonas syringae pv tomato DC3000 in infected tomato leaves. Mol Plant Pathol.

[CR8] Buell CR, Joardar V, Lindeberg M, Selengut J, Paulsen IT, Gwinn ML, Dodson RJ, Deboy RT, Durkin AS, Kolonay JF, Madupu R (2003). The complete genome sequence of the Arabidopsis and tomato pathogen Pseudomonas syringae pv tomato DC3000. Proc Natl Acad Sci USA.

[CR9] Chung IY, Sim N, Cho YH (2012). Antibacterial efficacy of temperate phage-mediated inhibition of bacterial group motilities. Antimicrob Agents Chemother.

[CR10] Clavijo-Coppens F, Ginet N, Cesbron S, Briand M, Jacques MA, Ansaldi M (2021). Novel virulent bacteriophages infecting mediterranean isolates of the plant pest *Xylella*
*fastidiosa* and *Xanthomonas*
*albilineans*. Viruses.

[CR11] Conlin KC, Mccarter SM (1983). Effectiveness of selected chemicals in inhibiting Pseudomonas syringae pv tomato in vitro and in controlling bacterial speck. Plant Dis.

[CR12] Cuppels DA, Elmhirst J (1999). Disease development and changes in the natural *Pseudomonas*
*syringae* pv. *tomato* populations on field tomato plants. Plant Dis.

[CR13] de Jonge PA, Nobrega FL, BrounsBE,  SJD (2019). Molecular and evolutionary determinants of bacteriophage host range. Tr Microbiol.

[CR14] De Smet J, Hendrix H, Blasdel BG, Danis-Wlodarczyk K, Lavigne R (2017). *Pseudomonas* predators: understanding and exploiting phage–host interactions. Nat Rev Microbiol.

[CR15] Delcher A (1999). Improved microbial gene identification with GLIMMER. Nucleic Acids Res Spec Publ.

[CR16] Di Lallo G, Matteo E, Francesco M (2014) Isolation and Partial Characterization of Bacteriophages InfectingPseudomonas Syringaepv.actinidiae, Causal Agent of Kiwifruit Bacterial Canker. J Basic Microbiol 54(11):1210–1221. 10.1002/jobm.201300951 10.1002/jobm.201300951 24810619

[CR17] Durairaj K, Velmurugan P, Park JH, Chang WS, Park YJ, Senthilkumar P, Choi KM, Lee JH, Oh BT (2018). Characterization and assessment of two biocontrol bacteria against *Pseudomonas*
*syringae* wilt in *Solanum*
*lycopersicum* and its genetic responses. Microbiol Res.

[CR18] Fatouros G, Gkizi D, Fragkogeorgi GA, Paplomatas EJ, Tjamos SE (2017). Biological control of *Pythium, Rhizoctonia* and *Sclerotinia* in lettuce: association of the plant protective activity of the bacterium *Paenibacillus*
*alvei* K165 with the induction of systemic resistance. Plant Pathol J.

[CR19] Flores O, Retamales J, Núñez M, León M, Salinas P, Besoain X, Yañez C, Bastías R (2020). Characterization of bacteriophages against Pseudomonas syringae pv actinidiae with potential use as natural antimicrobials in kiwifruit plants. Microorganisms.

[CR20] Ghods S, Sims IM, Moradali MF, Rehm BHA (2015). Bactericidal compounds controlling growth of the plant pathogen Pseudomonas syringae pv actinidiae, which forms biofilms composed of a novel exopolysaccharide. Appl Environ Microbiol.

[CR21] Gkizi D, Lehmann S, L’Haridon F, Serrano M, Paplomatas EJ, Métraux JP, Tjamos SE (2016). The innate immune signaling system as a regulator of disease resistance and induced systemic resistance activity against *Verticillium*
*dahliae*. Mol Plant Microbe Interact.

[CR22] Griffin K, Campbell P. Gambley, C,  (2019). Genetic basis of copper-tolerance in Australian Pseudomonas syringae pv tomato. Australasian Plant Pathol.

[CR23] Gupta S Roy A, (2018) Plant cell wall: a simple physical barrier or a complex defense modulator–exploring its dynamic role at plant-fungus interface. Molecular Aspects of Plant-Pathogen Interaction, pp.333–351

[CR24] Gullino ML, Gilardi G, Sanna M et al. (2009) Epidemiology of Pseudomonas syringae pv. syringae on tomato. Phytoparasitica 37:461–466. 10.1007/s12600-009-0055-2

[CR25] Herman MAB, Davidson JK, Smart CD (2008). Induction of plant defense gene expression by plant activators and Pseudomonas syringae pv tomato in greenhouse-grown tomatoes. Phytopathology.

[CR26] Holtappels D, Fortuna K, Lavigne R, Wagemans J (2021). The future of phage biocontrol in integrated plant protection for sustainable crop production. Curr Opin Biotechnol.

[CR27] Holtappels D, Fortuna KJ, Moons L, Broeckaert N, Bäcker LE, Venneman S, Rombouts S, Lippens L, Baeyen S, Pollet S, Noben JP, Oechslin F, Vallino M, Aertsen A, Maes M, Van JV, Lavigne R, Wagemans J (2022). The potential of bacteriophages to control Xanthomonas campestris pv campestris at different stages of disease development. Microb Biotechnol.

[CR28] Ji P, Campbell HL, Kloepper JW, Jones JB, Suslow TV, Wilson M (2006). Integrated biological control of bacterial speck and spot of tomato under field conditions using foliar biological control agents and plant growth-promoting rhizobacteria. Biol Control.

[CR29] Jones JB, Jackson LE, Balogh B, Obradovic A, Iriarte FB, Momol MT (2007). Bacteriophages for plant disease control. Annu Rev Phytopathol.

[CR30] Jones JB, Vallad GE, Iriarte FB, Obradović A, Wernsing MH, Jackson LE, Balogh B, Hong JC, Momol MT (2012). Considerations for using bacteriophages for plant disease control. Bacteriophage.

[CR31] Kearse M, Moir R, Wilson A, Stones-Havas S, Cheung M, Sturrock S, Buxton S, Cooper A, Markowitz S, Duran C, Thierer T, Ashton B, Meintjes P, Drummond A (2012). Geneious basic: an integrated and extendable desktop software platform for the organization and analysis of sequence data. Bioinform.

[CR32] Krupovic M, Koonin EV (2017). Multiple origins of viral capsid proteins from cellular ancestors. Proc Natl Acad Sci USA.

[CR33] Krupovic M, Dutilh BE, Adriaenssens EM, Wittmann J, Vogensen FK, Sullivan MB, Rumnieks J, Prangishvili D, Lavigne R, Kropinski AM, Klumpp J, Gillis A, Enault F, Edwards RA, Duffy S, Clokie MRC, Barylski J, Ackermann HW, Kuhn JH (2016). Taxonomy of prokaryotic viruses: update from the ICTV bacterial and archaeal viruses subcommittee. Arch Virol.

[CR34] Kwan T, Liu J, DuBow M, Gros P, Pelletier J (2006). Comparative genomic analysis of 18 *Pseudomonas*
*aeruginosa* bacteriophages. J Bacteriol.

[CR35] Low SJ, Džunková M, Chaumeil PA, Parks DHH, P,  (2019). Evaluation of a concatenated protein phylogeny for classification of tailed double-stranded DNA viruses belonging to the order *Caudovirales*. Nat Microbiol.

[CR36] Luke K, Radek A, Liu X, Campbell J, Uzan M, Haselkorn R, Kogan Y (2002). Microarray analysis of gene expression during bacteriophage T4 infection. Virol.

[CR37] Newman MA, Roepenack-Lahaye E, Parr A, Daniels MJ, Dow JM (2002). Prior exposure to lipopolysaccharide potentiates expression of plant defenses in response to bacteria. Plant J.

[CR38] Ni P, Wang L, Deng B, Jiu S, Ma C, Zhang C, Almeida A, Wang D, Xu W, Wang S (2021). Characterization of a lytic bacteriophage against Pseudomonas syringae pv actinidiae and its endolysin. Viruses.

[CR39] Nishimura Y, Yoshida T, Kuronishi M, Uehara H, Ogata H, Goto S (2017). ViPTree: the viral proteomic tree server. Bioinformatics.

[CR40] Niu DD, Wang CJ, Guo YH, Jiang CH, Zhang WZ, Wang Y, Guo JH (2012). The plant growth-promoting rhizobacterium *Bacillus*
*cereus* AR156 induces resistance in tomato with induction and priming of defence response. Biocontrol Sci Technol.

[CR41] Overbeek R, Olson R, Pusch GD, Olsen GJ, Davis JJ, Disz T, Edwards RA, Gerdes S, Parrello B, Shukla M, Vonstein V, Wattam AR, Xia F, Stevens R (2013). The SEED and the rapid annotation of microbial genomes using subsystems technology (RAST). Nucleic Acids Res Spec Publ.

[CR42] O’Toole George A, Roberto K (1998) Initiation of Biofilm Formation InPseudomonas FluorescensWCS365 Proceeds via Multiple, Convergent Signalling Pathways: A Genetic Analysis. Mol Microbiol 28(3):449–461. 10.1046/j.1365-2958.1998.00797.x 10.1046/j.1365-2958.1998.00797.x 9632250

[CR43] Pinheiro LAM, Pereira C, Frazão C, Balcão VM, Almeida A (2019). efficiency of phage φ6 for biocontrol of Pseudomonas syringae pv syringae: an in vitro preliminary study. Microorganisms.

[CR44] Preston GM (2000). Pseudomonas syringae pv tomato: the right pathogen, of the right plant, at the right time. Mol Plant Pathol.

[CR45] Ramakers C, Ruijter JM, Deprez RH, Lekanne MAFM (2003). Assumption-free analysis of quantitative real-time polymerase chain reaction (PCR) data. Neurosci Lett.

[CR46] Safaie-Farahani A, Taghavi SM (2017). Transcript analysis of some defense genes of tomato in response to host and non-host bacterial pathogens. Mol Biol Res Commun.

[CR47] Scheck HJ, Pscheidt JW, Moore LW (1996). Copper and streptomycin resistance in strains of *Pseudomonas*
*syringae* from Pacific Northwest nurseries. Plant Dis.

[CR48] Schneider CA, Rasband WS, Eliceiri KW (2012). NIH Image to ImageJ: 25 years of image analysis. Nat Methods.

[CR49] Skliros D, Kalatzis PG, Katharios P, Flemetakis E (2016). Comparative functional genomic analysis of two vibrio phages reveals complex metabolic interactions with the host cell. Front Microbiol.

[CR50] Skliros D, Karpouzis E, Kalloniati C, Katharios P, Flemetakis E (2022). Comparative genomic analysis of dwarf *Vibrio* myoviruses defines a conserved gene cluster for successful phage infection. Arch Virol.

[CR51] Stefani E, Obradović A, Gašić K, Altin I, Nagy IK, Kovács T (2021). Bacteriophage-mediated control of phytopathogenic Xanthomonads: a promising green solution for the future. Microorganisms.

[CR52] Sundin GW, Wang N (2018). Antibiotic resistance in plant-pathogenic bacteria. Annu Rev Phytopathol.

[CR53] Szermer-Olearnik B, Boratyński J (2015). Removal of endotoxins from bacteriophage preparations by extraction with organic solvents. PLoS One.

[CR54] Tameshige T, Fujita H, Watanabe K, Toyokura K, Kondo M, Tatematsu K, Matsumoto N, Tsugeki R, Kawaguchi M, Nishimura M, Okada K (2013). Pattern dynamics in adaxial-abaxial specific gene expression are modulated by a plastid retrograde signal during *Arabidopsis*
*thaliana* leaf development. PLoS Genet.

[CR55] Tarakanov RI, Lukianova AA, Evseev PV, Toshchakov SV, Kulikov EE, Ignatov AN, Miroshnikov KA, Dzhalilov FSU (2022). Bacteriophage control of Pseudomonas savastanoi pv glycinea in soybean. Plants.

[CR56] Weber PH, Bujarski JJ (2015). Multiple functions of capsid proteins in (+) stranded RNA viruses during plant–virus interactions. Virus Res.

[CR57] Wilson M, Campbell HL, Ji P, Jones JB, Cuppels DA (2002). Biological control of bacterial speck of tomato under field conditions at several locations in North America. Phytopathology.

[CR58] Xin XF, He SY (2013). Pseudomonas syringae pv tomato DC 3000: a model pathogen for probing disease susceptibility and hormone signaling in plants. Annu Rev Phytopathol.

[CR59] Yin Y, Ni P, Deng B, Wang S, Xu W, Wang D (2018). Isolation and characterisation of phages against Pseudomonas syringae pv actinidiae Acta Agric Scand - B Soil. Plant Sci.

[CR60] Yu JG, Lim JA, Song YR, Heu S, Kim GH, Koh YJ, Oh CS (2016). Isolation and characterization of bacteriophages against Pseudomonas syringae pv actinidiae causing bacterial canker disease in kiwifruit. J Microbiol Biotechnol.

[CR61] Zaccardelli M, Spasiano A, Bazzi C, Merighi M (2005). Identification and in planta detection of Pseudomonas syringae pv tomato using PCR amplification of hrpZPst. Eur J Plant Pathol.

[CR62] Żaczek M, Weber-Dąbrowska B, Górski A (2015). Phages in the global fruit and vegetable industry. J Appl Microbiol.

[CR63] Zafar N, Mazumder R, Seto D (2002). CoreGenes: a computational tool for identifying and cataloging ‘core’ genes in a set of small genomes. Eur J Plant Pathol.

[CR64] Zhang H, Wu H, Xia H, Zhong C, Li L, Zeng C (2022). Genomic characterization of two nickie-like bacteriophages that infect the kiwifruit canker phytopathogen Pseudomonas syringae pv actinidiae. Arch Virol.

