# Supplementary material for: In planta interactions of a novel bacteriophage against Pseudomonas syringae pv. tomato
Source: Appl Microbiol Biotechnol. 2023 Apr 19;107(11):3801–15. doi: 10.1007/s00253-023-12493-5 (PMC10175458; doi:10.1007/s00253-023-12493-5)
Supplement: Supplementary file 1 — Supplementary file1 (PDF 1123 KB) [file 253_2023_12493_MOESM1_ESM.pdf]

In planta interactions of a novel bacteriophage against *Pseudomonas syringae* pv. *tomato*

Dimitrios Skliros[1], Polyxeni Papazoglou[1], Danai Gkizi[2], Elena Paraskevopoulou[1], Pantelis Katharios[3], Dimitrios Goumas[4], Sotirios Tjamos[5*], Emmanouil Flemetakis[1*]

1. Laboratory of Molecular Biology, Department of Biotechnology, School of Applied Biology and Biotechnology, Agricultural University of Athens, 11855 Athens, Greece
2. Department of Wine, Vine and Beverage Sciences, School of Food Sciences, University of West Attica, 12243 Athens Greece
3. Institute of Marine Biology, Biotechnology and Aquaculture, Hellenic Centre for Marine Research, 71500 Heraklion, Greece
4. Laboratory of Plant Pathology-Bacteriology, Department of Agriculture, School of Agricultural Sciences, Hellenic Mediterranean University, Estavromenos, 71004 Heraklio, Greece
5. Laboratory of Plant Pathology, Department of Crop Science, School of Plant Sciences, Agricultural University of Athens, 1855 Athens, Greece

*** Address correspondence to:**

*Sotirios Tjamos, Laboratory of Plant Pathology, Department of Crop Science, School of Plant Sciences, Agricultural University of Athens, 1855 Athens, Greece, sotiris@aua.gr, 00302105294519

*Emmanouil Flemetakis, Laboratory of Molecular Biology, Department of Biotechnology, School of Applied Biology and Biotechnology, Agricultural University of Athens, 11855 Athens, Greece, mflem@aua.gr, 00302105294343

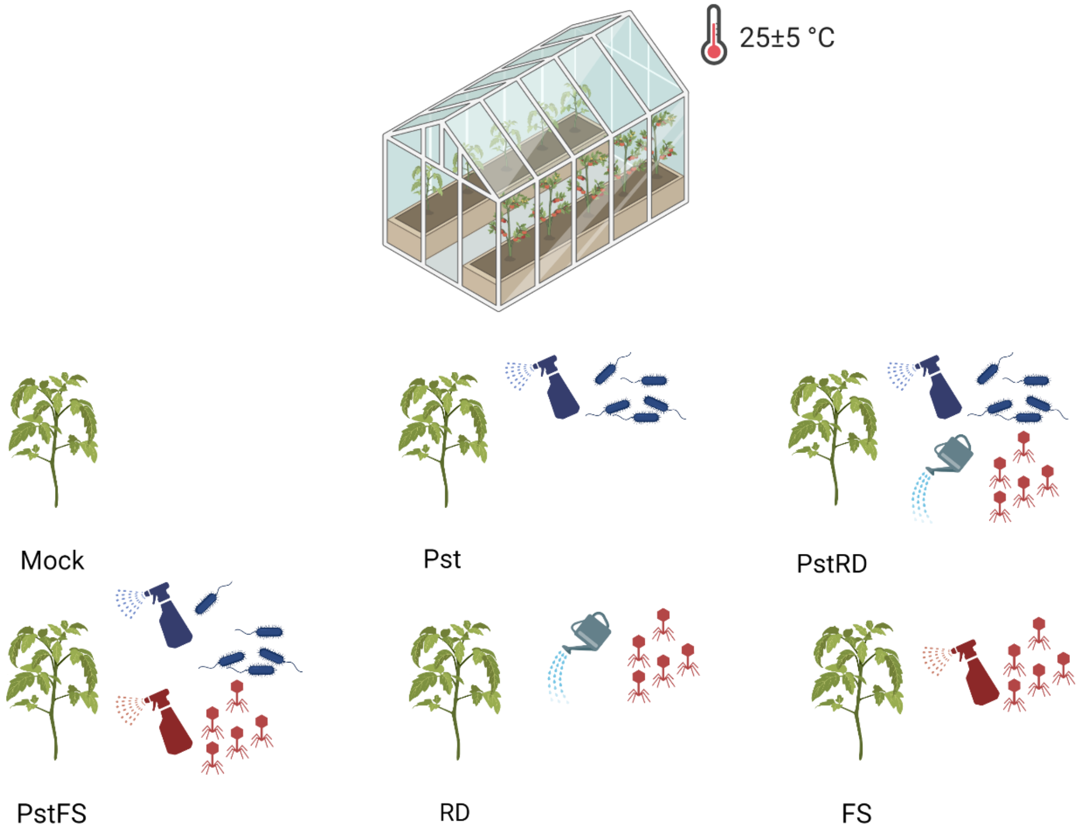

Fig. S1 Schematic representation of the two application strategies, one by root drenching with a phage-based solution and one by foliar spraying, both 24 hours prior infecting with Pst. Mock→mock plants, Pst→*Pseudomoans syringae* application, PstRD→ phage root drenching method with *P. syringae* application, PstFS phage foliar spraying method *P. syringae* application, RD→ phage root drenching method solely, FS→ phage foliar spraying method solely.

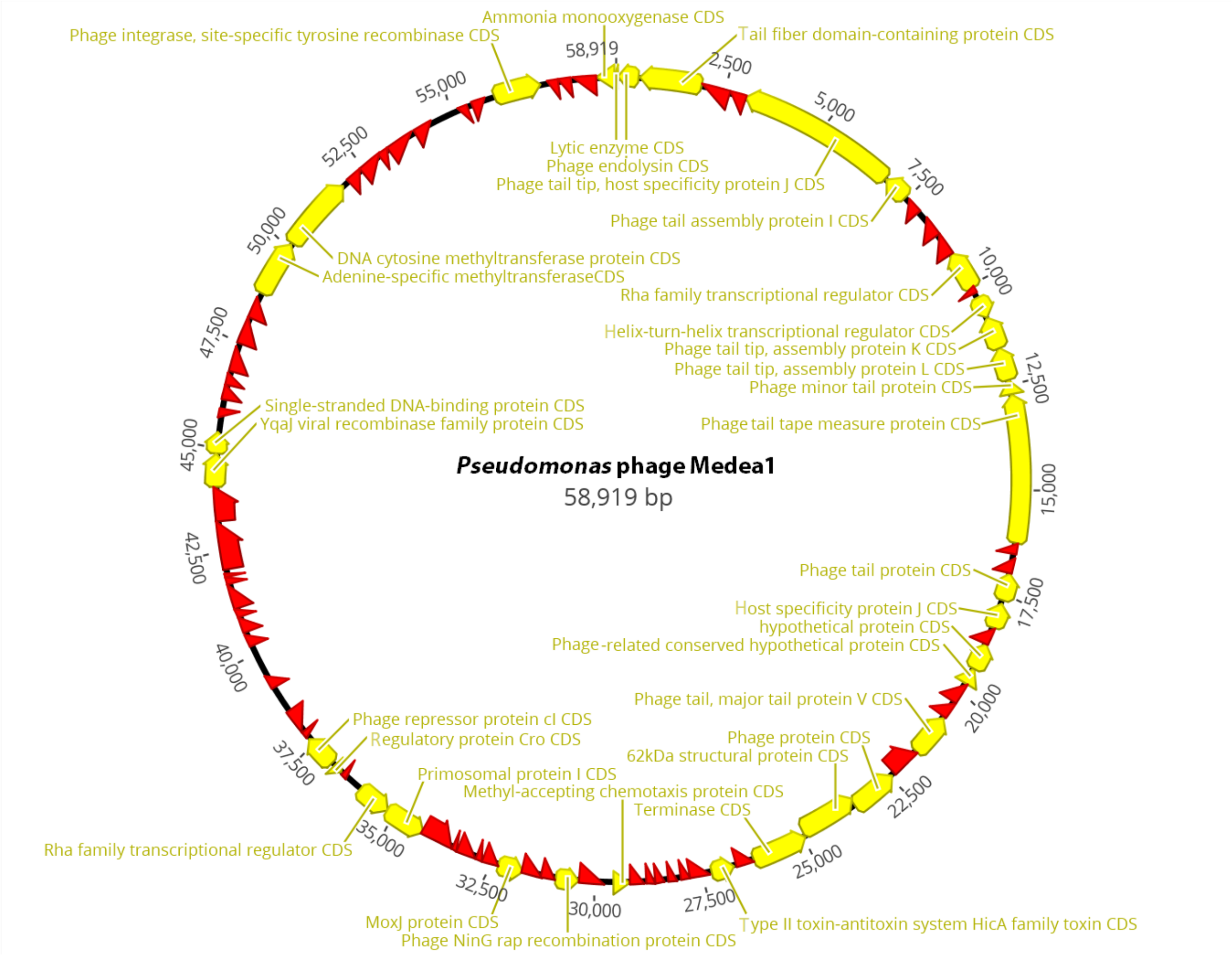

Fig. S2 Circular schematic representation created with Geneious software (R10 version) of the genome of bacteriophage Medea1 including annotations. Yellow arrows correspond to CDSs with known function and red arrows correspond to hypothetical proteins.

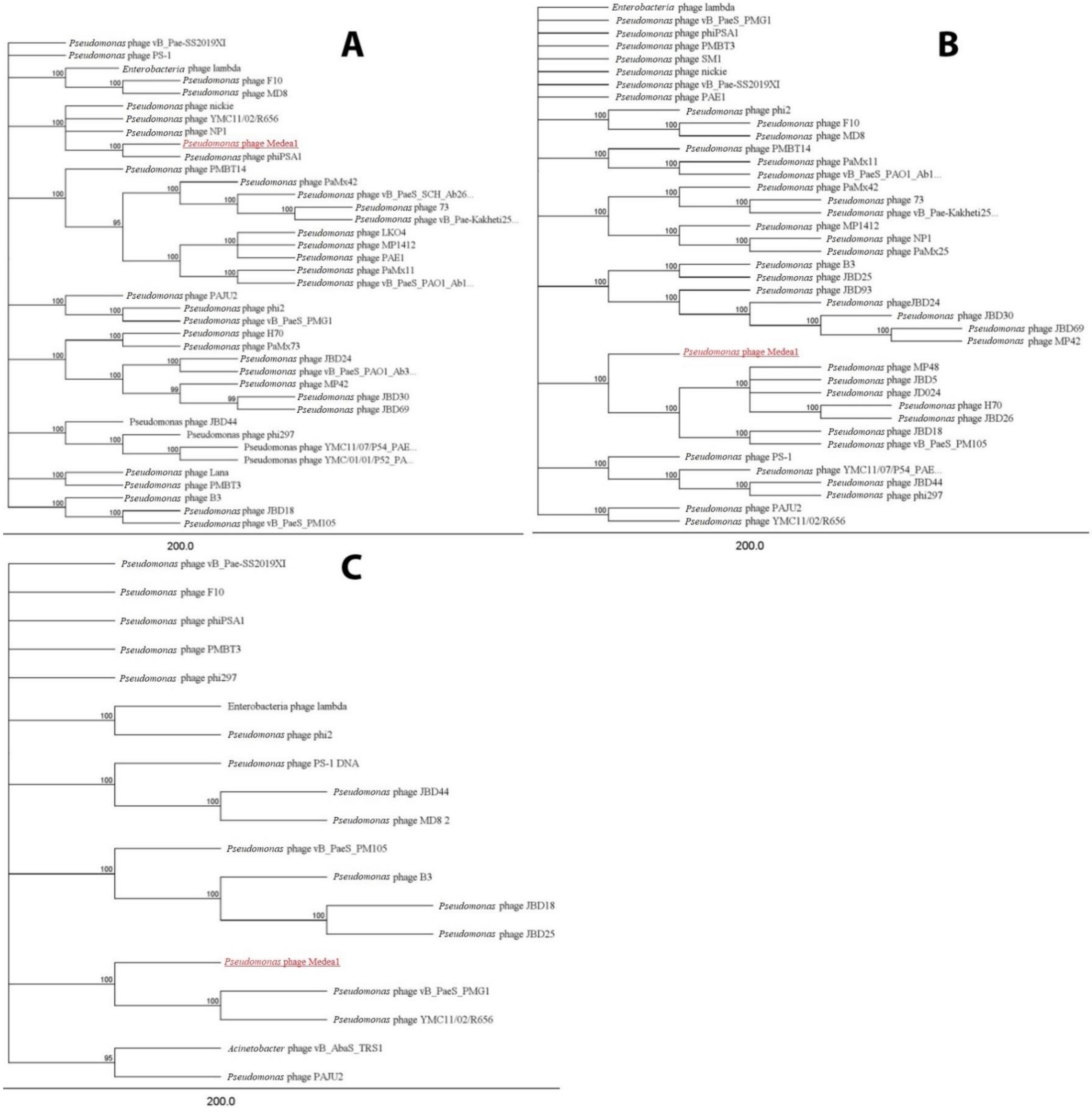
A
Pseudomonas phage vB_Pae-SS2019XI
Pseudomonas phage PS-1
Enterobacteria phage lambda
Pseudomonas phage F10
Pseudomonas phage MD8
Pseudomonas phage nickie
Pseudomonas phage YMC11/02/R656
Pseudomonas phage NP1
Pseudomonas phage Medea1
Pseudomonas phage phiPSA1
Pseudomonas phage PMBT14
Pseudomonas phage PaMx42
Pseudomonas phage vB_PaeS_SCH_Ab26...
Pseudomonas phage 73
Pseudomonas phage vB_Pae-Kakheti25...
Pseudomonas phage LKO4
Pseudomonas phage MP1412
Pseudomonas phage PAE1
Pseudomonas phage PaMx11
Pseudomonas phage vB_PaeS_PAO1_Ab1...
Pseudomonas phage PAJU2
Pseudomonas phage phi2
Pseudomonas phage vB_PaeS_PMG1
Pseudomonas phage H70
Pseudomonas phage PaMx73
Pseudomonas phage JBD24
Pseudomonas phage vB_PaeS_PAO1_Ab3...
Pseudomonas phage MP42
Pseudomonas phage JBD30
Pseudomonas phage JBD69
Pseudomonas phage JBD44
Pseudomonas phage phi297
Pseudomonas phage YMC11/07/P54_PAE...
Pseudomonas phage YMC/01/01/P52_PA...
Pseudomonas phage Lana
Pseudomonas phage PMBT3
Pseudomonas phage B3
Pseudomonas phage JBD18
Pseudomonas phage vB_PaeS_PM105
200.0
B
Enterobacteria phage lambda
Pseudomonas phage vB_PaeS_PMG1
Pseudomonas phage phiPSA1
Pseudomonas phage PMBT3
Pseudomonas phage SM1
Pseudomonas phage nickie
Pseudomonas phage vB_Pae-SS2019XI
Pseudomonas phage PAE1
Pseudomonas phage phi2
Pseudomonas phage F10
Pseudomonas phage MD8
Pseudomonas phage PMBT14
Pseudomonas phage PaMx11
Pseudomonas phage vB_PaeS_PAO1_Ab1...
Pseudomonas phage PaMx42
Pseudomonas phage 73
Pseudomonas phage vB_Pae-Kakheti25...
Pseudomonas phage MP1412
Pseudomonas phage NP1
Pseudomonas phage PaMx25
Pseudomonas phage B3
Pseudomonas phage JBD25
Pseudomonas phage JBD93
Pseudomonas phageJBD24
Pseudomonas phage JBD30
Pseudomonas phage JBD69
Pseudomonas phage MP42
Pseudomonas phage Medea1
Pseudomonas phage MP48
Pseudomonas phage JBD5
Pseudomonas phage JD024
Pseudomonas phage H70
Pseudomonas phage JBD26
Pseudomonas phage JBD18
Pseudomonas phage vB_PaeS_PM105
Pseudomonas phage PS-1
Pseudomonas phage YMC11/07/P54_PAE...
Pseudomonas phage JBD44
Pseudomonas phage phi297
Pseudomonas phage PAJU2
Pseudomonas phage YMC11/02/R656
200.0
C
Pseudomonas phage vB_Pae-SS2019XI
Pseudomonas phage F10
Pseudomonas phage phiPSA1
Pseudomonas phage PMBT3
Pseudomonas phage phi297
Enterobacteria phage lambda
Pseudomonas phage phi2
Pseudomonas phage PS-1 DNA
Pseudomonas phage JBD44
Pseudomonas phage MD8 2
Pseudomonas phage vB_PaeS_PM105
Pseudomonas phage B3
Pseudomonas phage JBD18
Pseudomonas phage JBD25
Pseudomonas phage Medea1
Pseudomonas phage vB_PaeS_PMG1
Pseudomonas phage YMC11/02/R656
Acinetobacter phage vB_AbaS_TRS1
Pseudomonas phage PAJU2
200.0

Fig. S3. Phylogenetic trees of three amino acid sequences of *Pseudomonas* phages including *Enterobacteria* phage lambda. Tail-tape measure protein **(A)** of bacteriophages SM1 (YP_009594421), PMBT3 (YP_009796565), phiPSA1 (YP_009043554), vB_PaeS_PM105 (YP_009188557), PaMx42 (YP_009205616), JBD18 (YP_009168321), JBD93 (YP_009274633), JBD25 (YP_009168771), vB_Pae-Kakheti25 (YP_006299885), MP48 (YP_009055269), JD024 (YP_009042226), JBD26 (AEY99434), H70 (YP_009152351), JBD5 (YP_007392753), Nickie (YP_009620693), JBD24 (YP_007392811), MP42 (YP_006560541), JBD69 (YP_009273672), JBD30 (YP_007392353), Medea1 (QVW29083), B3 (YP_164086), PS-1 (YP_009222841), YMC11/02/R656 (YP_009187461), phi297 (YP_005098083), JBD44 (YP_009275520), PaMx25 (YP_009603571), NP1 (YP_009285831), MP1412 (YP_006561076), PAJU2 (YP_002284352), vB_PaeS_PMG1 (YP_005098222), YMC11/07/P54_PAE_BP (YP_009273705), PMBT14 (YP_009836195), MD8 (YP_009289244), F10 (YP_001293357), vB_PaeS_PAO1_Ab19, PaMx11 (YP_009196278), phi2 (YP_009275675), PAE1 (YP_009215757), vB_Pae-SS2019XI (YP_010000113), and *Enterobacteria* phage lambda (NP_040595). Terminase large subunit **(B)** of bacteriophages vB_Pae-SS2019XI (YP_010000103), F10 (YP_001293346), MD8 (YP_009289232), Lana (YP_009820332), PMBT3 (YP_009796552), phi2 (YP_009275664), vB_PaeS_PM105 (YP_009188542), JBD18 (YP_009168307), vB_PaeS_PMG1 (YP_005098205), PaMx73 (AII21875), H70 (YP_009152333), vB_PaeS_PAO1_Ab30 (YP_009125671), MP42 (YP_006560525), JBD69 (YP_009273657), JBD30 (YP_007392338), JBD24 (YP_007392794), PaMx42 (YP_009205602), vB_PaeS_SCH_Ab26 (YP_009044341), vB_Pae-Kakheti25 (YP_006299871), 73 (YP_001293413), PAE1 (YP_009215693), MP1412 (YP_006561058), LKO4 (YP_009601848), PAJU2 (YP_002284336), PMBT14 (YP_009836182), vB_PaeS_PAO1_Ab19 (YP_009966481), PaMx11 (YP_009196255), PS-1 (YP_009222820), B3 (YP_164067), NP1 (YP_009285816), YMC11/02/R656 (YP_009187477), Medea1 (QVW29097), Nickie (YP_009620680), phiPSA1 (YP_009043593), JBD44 (YP_009275505), YMC/01/01/P52_PAE_BP (AFQ22045), YMC11/07/P54_PAE_BP (YP_005098068), and *Enterobacteria* phage lambda (NP_040581) and Integrase protein **(C)** of bacteriophages JBD25 (YP_009168739), JBD18 (YP_009168291), B3 (YP_164048), vB_PaeS_PM105 (YP_009188523), JBD44 (YP_009275533), PS-1 (YP_009222771), (YP_009043567), MD82 (YP_009289261), phi297 (YP_005098095), YMC11/02/R656 (YP_009187432), vB_PaeS_PMG1 (YP_005098238), phi2 (YP_009275635), Medea1 (QVW29150), F10 (YP_001293372), vB_Pae-SS2019XI (YP_010000184), PAJU2

(YP_002284365), PMBT3 (YP_009796665), *Enterobacteria* phage lambda (NP_040609), and *Acinetobacter* phage vB_AbaS_TRS1 (YP_009289773).

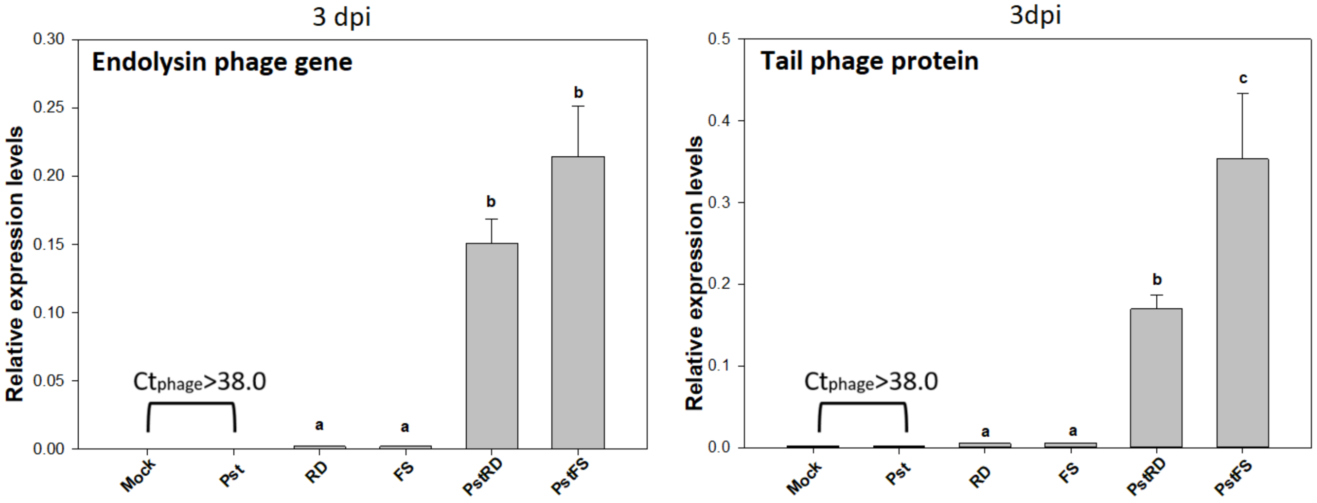

Fig. S4. Relative expression levels analysis of *Pseudomonas* phage Medea1 ORFs referring to tail-phage transcripts (QVW29086) and phage endolysin transcripts gene (QVW29068) in tomato leaves 3 days post-inoculation. Pst for application without bacteriophage, RD for root drenching application of phage without Pst, FS for foliar spray application of phage without Pst, PstRD for root drenching application with Pst, PstFS for foliar spraying application with Pst. Different letters indicate statistically significant differences among treatments (SE ±, ANOVA p<0.05 post-hoc student's t-test, n=3).

Supplemental Table S1. Putative genes of Medea1 and their protein homologies compared to *Enterobacteria* phage lamda and *Pseudomonas* phage PSA1 bacteriophages. ✓ symbol shows presence of that protein with at least a blastp threshold score of 45.

Protein ID	Size (aa)	Putative function	Presence to lambda	Presence to PSA1
**QVW29068.1**	177	Endolysin		✓
**QVW29069.1**	477	Tail fiber domain-containing protein		✓
**QVW29070.1**	220	Hypothetical protein		✓
**QVW29071.1**	122	Hypothetical protein		✓
**QVW29072.1**	1268	Host specificity protein J	✓	✓
**QVW29073.1**	197	Tail assembly protein I	✓	
**QVW29074.1**	130	Hypothetical protein		
**QVW29075.1**	152	Hypothetical protein		
**QVW29076.1**	191	Hypothetical protein		
**QVW29077.1**	307	Rha family transcriptional regulator	✓	
**QVW29078.1**	54	Hypothetical protein		
**QVW29079.1**	169	Helix-turn-helix transcriptional regulator	✓	✓
**QVW29080.1**	254	Assembly protein K	✓	✓
**QVW29081.1**	249	Assembly protein L	✓	
**QVW29082.1**	112	Minor tail protein	✓	
**QVW29083.1**	1157	Tail tape measure protein	✓	✓
**QVW29084.1**	82	Hypothetical protein		
**QVW29085.1**	127	Hypothetical protein		
**QVW29086.1**	218	Tail protein	✓	
**QVW29087.1**	204	Specificity protein J	✓	✓
**QVW29088.1**	186	Hypothetical protein		
**QVW29089.1**	218	Hypothetical protein		✓
**QVW29090.1**	122	Hypothetical protein		✓
**QVW29091.1**	166	Hypothetical protein		✓
**QVW29092.1**	142	Hypothetical protein		
**QVW29093.1**	316	Major tail protein V	✓	
**QVW29094.1**	242	Hypothetical protein		
**QVW29095.1**	361	Minor capsid protein		✓

**QVW29096.1**	473	62kda structural protein		
**QVW29097.1**	433	Terminase		✓
**QVW29098.1**	153	Hypothetical protein		
**QVW29099.1**	187	Toxin		✓
**QVW29100.1**	159	Hypothetical protein		✓
**QVW29101.1**	65	Hypothetical protein		
**QVW29102.1**	66	Hypothetical protein	✓	
**QVW29103.1**	58	Hypothetical protein		
**QVW29104.1**	72	Hypothetical protein		
**QVW29105.1**	112	Hypothetical protein	✓	✓
**QVW29106.1**	123	Methyl-accepting chemotaxis protein		✓
**QVW29107.1**	193	Hypothetical protein		✓
**QVW29108.1**	196	Recombination protein	✓	✓
**QVW29109.1**	104	Hypothetical protein	✓	
**QVW29110.1**	156	Hypothetical protein	✓	
**QVW29111.1**	188	Moxj protein		
**QVW29112.1**	134	Hypothetical protein		
**QVW29113.1**	62	Hypothetical protein	✓	
**QVW29114.1**	149	Hypothetical protein		
**QVW29115.1**	51	Hypothetical protein		
**QVW29116.1**	261	Hypothetical protein	✓	
**QVW29117.1**	326	Primosomal protein I		
**QVW29118.1**	268	Rha family transcriptional regulator	✓	✓
**QVW29119.1**	63	Hypothetical protein		
**QVW29120.1**	68	Regulatory protein Cro	✓	
**QVW29121.1**	248	Repressor protein ci	✓	
**QVW29122.1**	51	Hypothetical protein		
**QVW29123.1**	187	Hypothetical protein		
**QVW29124.1**	111	Hypothetical protein		✓
**QVW29125.1**	99	Hypothetical protein		
**QVW29126.1**	135	Hypothetical protein		
**QVW29127.1**	64	Hypothetical protein		

**QVW29128.1**	199	Hypothetical protein		✓
**QVW29129.1**	56	Hypothetical protein		
**QVW29130.1**	38	Hypothetical protein		
**QVW29131.1**	387	Hypothetical protein	✓	✓
**QVW29132.1**	271	Hypothetical protein	✓	
**QVW29133.1**	255	Viral recombinase family protein	✓	
**QVW29134.1**	163	Single-stranded DNA-binding protein		✓
**QVW29135.1**	71	Hypothetical protein		
**QVW29136.1**	115	Hypothetical protein		✓
**QVW29137.1**	103	Hypothetical protein		
**QVW29138.1**	203	Hypothetical protein		
**QVW29139.1**	206	Hypothetical protein		✓
**QVW29140.1**	164	Hypothetical protein		
**QVW29141.1**	430	Adenine-specific methyltransferase		✓
**QVW29142.1**	600	DNA cytosine methyltransferase protein		
**QVW29143.1**	130	Hypothetical protein		
**QVW29144.1**	192	Hypothetical protein	✓	
**QVW29145.1**	75	Hypothetical protein		
**QVW29146.1**	232	Hypothetical protein		
**QVW29147.1**	145	Hypothetical protein		
**QVW29148.1**	70	Hypothetical protein		
**QVW29149.1**	103	Hypothetical protein		
**QVW29150.1**	363	Integrase	✓	✓
**QVW29151.1**	91	Hypothetical protein		✓
**QVW29152.1**	98	Hypothetical protein		
**QVW29153.1**	181	Hypothetical protein		✓
**QVW29154.1**	137	Ammonia monooxygenase		
